# From Classic to Modern Prognostic Biomarkers in Patients with Acute Myocardial Infarction

**DOI:** 10.3390/ijms23169168

**Published:** 2022-08-15

**Authors:** Cristian Stătescu, Larisa Anghel, Bogdan-Sorin Tudurachi, Andreea Leonte, Laura-Cătălina Benchea, Radu-Andy Sascău

**Affiliations:** 1 Cardiology Department, Cardiovascular Diseases Institute “Prof. Dr. George I. M. Georgescu”, 700503 Iași, Romania; 2 Internal Medicine Department, “Grigore T. Popa” University of Medicine and Pharmacy, 700503 Iași, Romania

**Keywords:** biomarkers, myocardial infarction, prognosis, cardiac function

## Abstract

Despite all the important advances in its diagnosis and treatment, acute myocardial infarction (AMI) is still one of the most prominent causes of morbidity and mortality worldwide. Early identification of patients at high risk of poor outcomes through the measurement of various biomarker concentrations might contribute to more accurate risk stratification and help to guide more individualized therapeutic strategies, thus improving prognoses. The aim of this article is to provide an overview of the role and applications of cardiac biomarkers in risk stratification and prognostic assessment for patients with myocardial infarction. Although there is no ideal biomarker that can provide prognostic information for risk assessment in patients with AMI, the results obtained in recent years are promising. Several novel biomarkers related to the pathophysiological processes found in patients with myocardial infarction, such as inflammation, neurohormonal activation, myocardial stress, myocardial necrosis, cardiac remodeling and vasoactive processes, have been identified; they may bring additional value for AMI prognosis when included in multi-biomarker strategies. Furthermore, the use of artificial intelligence algorithms for risk stratification and prognostic assessment in these patients may have an extremely important role in improving outcomes.

## 1. Introduction

Cardiovascular diseases are the leading cause of morbidity and mortality worldwide, with an estimated 17.9 million deaths annually [1]. Despite all of the important advancements in diagnosis and treatment, more than four out of five cardiovascular deaths are caused by acute myocardial infarction (AMI) and stroke [1,2]. Myocardial infarction is most often caused by a rupture or erosion of an atherosclerotic plaque involving acute atherothrombotic occlusion of an epicardial coronary artery [1]. The clinical definition of myocardial infarction relies on the presence of acute myocardial injury detected from abnormal cardiac biomarkers, especially cardiac troponin (cTn), in the identification of evidence for acute myocardial ischemia [3]. Myocardial infarction can be classified as ST-elevation myocardial infarction (STEMI) or non-ST-elevation myocardial infarction (NSTEMI), which determines the treatment strategy that is needed [1,3]. Patients with STEMI often have a higher incidence of transmural ischemia, a larger area of infraction and worse short-term prognosis in comparison with patients that present with NSTEMI. Reserved prognoses for patients with acute myocardial infarction involve a large area of infarction, development of left ventricular dysfunction and adverse remodeling [1]. Early identification of patients at high risk of poor outcomes through the measurement of various biomarker concentrations soon after the onset of MI might contribute to more accurate risk stratification in these patients. By assessing the risk for patients with acute myocardial infarction, therapeutic strategies can be tailored to each patient’s needs, thereby improving patients’ prognoses [4,5,6,7,8].

An ideal biomarker should have a high sensitivity and specificity, as well as several clinical and assay-related characteristics, such as a good cost-effectiveness ratio, short processing time and high precision. These characteristics ensure a straightforward assay process to guide therapy and predict the prognosis of patients. Currently, there is no single ideal biomarker that can improve risk prediction for patients with AMI; it is a considerable challenge for clinicians to discover an ideal biomarker. Thus, in order to identify novel predictive biomarkers, researchers have used proteomic technologies. Proteomics have been used to highlight the significant alterations in the cardiac proteome following an MI. A proteomic study of unstable plaques also revealed new evidence about the pathologic processes of plaque rupture [9]. This complex process involves changes at the cellular and molecular levels that impact ventricular size, shape and function after an AMI [10]. A study conducted by Shavadia et al. evaluated the associations between 24 h relative changes in the concentrations of 91 novel biomarkers and composite outcomes in 139 STEMI patients (72 cases, 67 controls) treated with primary percutaneous coronary intervention. The authors observed that the variations in the expression of 14 biomarkers linked with myocardial fibrosis and remodeling, inflammation, thrombosis and angiogenesis and cholesterol metabolism were substantially correlated with the outcomes within 90 days. Their results suggest the need to include synergistic multi-biomarker strategies for risk stratification in patients with AMI [11]. The use of multi-biomarker proteomics may also be combined with machine learning, as was undertaken for general cardiovascular risk by Williams et al. [12]. Machine-learning techniques can be used to design a protein model in order to determine the probability of outcomes of post-myocardial infarction.

**Figure 1:**
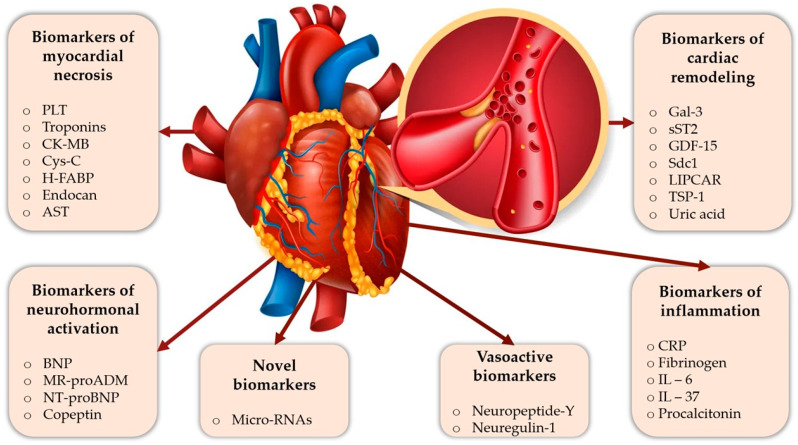
Prognostic biomarkers in patients with acute myocardial infarction. AST, aspartate transaminase; BNP, brain natriuretic peptide; CK-MB, creatine kinase-MB; cysC, cystatin C; CRP, C-reactive protein; Gal-3, galectin-3; GDF-15, growth differentiation factor-15; H-FABP, heart-type fatty acid binding protein; IL-6, interleukin-6; IL-37, interleukin-37; LIPCAR, a long noncoding ribonucleic acid; MicroRNAs, microribonucleic acids; MR-proADM, mid-regional proadrenomedullin; NT-proBNP, N terminal pro-brain natriuretic peptide; PLT, platelet; Sdc-1, syndecan-1; sST2, soluble suppression of tumorigenicity-2; TSP-1, thrombospondin-1.

Although there is no ideal biomarker that can provide prognostic information for risk assessment of patients with AMI, the results from recent years are promising [8,13,14]. For instance, several novel biomarkers have been identified that may bring additional value for AMI prognosis when included in multi-biomarker strategies. There is no generally accepted classification for these prognostic biomarkers; many of them fall into more than one category. To facilitate a better understanding, we decided to distribute them based on their pathogenesis as follows: biomarkers of inflammation, biomarkers of myocardial necrosis, neurohormonal activation biomarkers, cardiac remodeling biomarkers and vasoactive biomarkers (Figure 1).

The aim of this article is to provide an overview of the role and applications of cardiac biomarkers in risk stratification and prognostic assessment for patients with myocardial infarction. Additionally, this article reviews the classic biomarkers that have already demonstrated their prognostic role in patients with acute myocardial infarction, as well as new biomarkers that may have potential clinical value in the future.

## 2. Results

### 2.1. Biomarkers of Inflammation

#### 2.1.1. C-Reactive Protein

C-reactive protein (CRP) has become one of the most studied biomarkers of inflammation due to the fact that atherothrombosis represents a large majority of acute myocardial infarctions, and CRP is strongly associated with this type of pathogenesis. As an acute phase protein, CRP levels are elevated in inflammatory or infectious processes due to the liver’s response to high concentrations of plasma cytokines, such as interleukin-6 (IL-6) and tumor necrosis factor alpha (TNF-α). Increased levels of CRP have been reported as an independent predictive biomarker for cardiac or noncardiac death and even for recurrent nonfatal events, with detection of CRP at 4–6 h after the occurrence of AMI and a peak level of CRP after about 50 h [4,5]. Furthermore, CRP is directly involved in the generation of endothelial dysfunction in the process of atherosclerosis. Therefore, through the inhibition of the eNOS at the endothelial level, a decrease in nitric oxide synthesis results, which further leads to inhibition of angiogenesis, vasoconstriction and inflammation. In addition, CRP is part of the process of microvascular thrombus formation and, thus, microvascular obstruction through the development of leucocyte adhesion and platelet aggregation in the ischemic endothelium [6]. Usually, there is a strong inflammatory response after an AMI. This is defined by the following: secretion of cytokines and chemokine; expression of adhesion molecules on endothelial cells; activation of the complement cascade with secondary release of proinflammatory cytokines, which ultimately trigger myofibroblasts; and expression of matrix proteins, which play a protective role in the infarcted area [7]. CRP is an important biomarker in this case simply because it reflects the prolongation of the temporal and spatial inflammatory process. Hence, the higher the CRP value is, the larger the inflammatory response. To some degree, it may even reflect the size of the myocardial infarction area, which is strongly related to post-infarct left ventricular (LV) dysfunction [8,13]. Extremely elevated levels of CRP ≥ 20 mg/dL have been associated with severe inflammatory processes; as a result, CRP is a predictor value for ventricular aneurysm and subacute cardiac rupture [14]. Ming et al. [4] found that serum concentrations greater than 8.95 mg/L at admission are associated with cardiovascular, cardiac and long-term all-cause mortality after an AMI, regardless of the presence of diabetes mellitus. In a meta-analysis including seven studies (six retrospective studies and one cohort study), the elevated CRP group presented predictive values for greater risk of in-hospital and long-term mortality, major acute cardiovascular events (MACEs) and recurrent AMI, but without acute or subacute in-stent restenosis [15]. It has been postulated that every 1 mg/L in the CRP value correlates with a 12% increase in the risk of MACEs [13]. With the advancement of technology, much more sensitive determinations have been discovered, such as highly sensitive CRP (hs-CRP), which can detect much lower levels of CRP, around 0.5–1 mg/L compared to 5–20 mg/L in the case of classical moderately sensitive immunoassays for CRP [5]. There is scientific evidence that patients with hs-CRP values > 2 mg/L measured 30 days post-AMI (indicating persistent inflammatory response after the acute phase) have a two-fold increase in the risk of a new heart failure (HF) or of the worsening of an existing heart failure over the following 2 years [13,16]. At the same time, hs-CRP is not limited to predicting HF; it can also predict recurrent ischemic events and cardiovascular death. In a study conducted by Maarten et al. [16], it was found that only the peak CRP determined in the acute phase is statistically relevant for predicting left ventricular dysfunction (LVD) at follow-up. Christian et al. [17] demonstrated that peak CRP levels are a strong predictor for all-cause and cardiovascular mortality at one-year follow-up after ST-elevation myocardial infarction (STEMI). Moreover, CRP concentrations greater than 10 mg/L within 12 h after hospitalization predicted a 6% increase in the risk of poor evolution. This can imply left ventricular systolic dysfunction (LVSD) in short- and long-term follow-up, therefore suggesting an opposed correlation between in-admission CRP determination and left ventricle ejection fraction (LVEF) in multi-year follow-ups [13]. Iwona et al. [18] studied the prognostic value of CRP in patients with STEMI followed by percutaneous coronary intervention (PCI) and guideline-based treatment. Remarkably, there was a relation between persistently increased levels of CRP at admission and one month after discharge and an elevated risk for HF hospitalization and mortality in six years of follow-up. A single-center observational study [19] assessed the association between increased CRP and the risk for acute kidney injury (AKI) in STEMI patients treated with PCI; this study demonstrated that CRP is an independent marker for AKI in this category but with limitations in determining the adequate moment for measurement of its value during the 24 h after admission. Furthermore, Fu et al. [20] recently suggested that, in post-AMI patients treated according to guidelines, an increased hs-CRP ≥ 2 mg/L is related to later risk of AKI and progression of chronic kidney disease (CKD), regardless of baseline kidney function. In a prospective study, Ozgur et al. [21] demonstrated that the CRP to albumin ratio may also present potential usefulness as a predictor for poor clinical evolution in short-term follow-up of patients with STEMI, regardless of the number of vessels affected, but without precision in the assessment of the localization and width of the ischemic area.

#### 2.1.2. Fibrinogen

Fibrinogen (FIB) was one of the first clotting factors to be identified. It was first studied in the 19th century and represents an acute phase protein produced in the liver. Its effects are multiple, including on platelet aggregation, endothelial lesions, plasma viscosity and the fibrinolysis system, and it is therefore directly implicated in the formation of thrombi [22,23]. Through interactions with inflammatory cytokines, FIB has a significant role in the inflammatory response, impacting the onset and progression of cardiovascular disease. Furthermore, coronary artery restenosis can be provoked secondarily to FIB degradation products, which promotes smooth muscle cell proliferation [23]. Ang et al. [24] suggested that, in patients with acute coronary syndrome (ACS) after PCI, a higher baseline fibrinogen value (≥280 mg/dL) is correlated with an increased risk of MACEs within two years. These are defined as rehospitalization, revascularization, stent thrombosis, stroke or transient ischemic attack (TIA) and all-cause mortality. Earlier studies suggested cut-off values greater than 350 mg/dL for the same outcomes [25]. In addition, in patients with non-ST-elevation acute coronary syndrome (NSTE-ACS) treated with PCI, fibrinogen was an independent predictor of non-fatal infarction or death and was indistinguishable from the GRACE system score in anticipating such outcomes [23]. Some research has found that the fibrinogen to albumin ratio (FAR) has a more relevant link to clinical events than albumin and fibrinogen alone, being an independent predictor of MACEs in patients treated with PCI for ACS. The presence of albumin is not surprising, as it is a serum protein with important roles not only in the regulation of osmotic pressure in extracellular fluid but also in platelet aggregation through its increase of the production of prostaglandin D2. A low concentration of albumin may lead to endothelial dysfunction and was found to be an independent risk predictor for all-cause mortality at 40 months follow-up in patients with STEMI [25]. Being a feasible marker, as well as a cheap one, FAR could be considered a potential alternative tool to amplify the prognostic sensitivity value in patients with STEMI [26]. In addition, Liu et al. [27] studied the relationship between FAR and the SYNTAX score, demonstrating that there is a linear connection in this case, as well as benefits such as simplicity, efficiency and exhaustiveness. Furthermore, it has been suggested that high levels of pre-procedural FIB are an independent risk marker for contrast-induced kidney injury after catheterization in patients with ACS [23,28].

#### 2.1.3. Interleukin-6

Interleukin-6 (IL-6) is one of the most important cytokines. It is produced by T-cells and macrophages in response to a trauma or to an infection and involves hepatic CRP liberation in the second stage of the process [29]. It may also be considered as a pivot in inflammatory signaling during the atherosclerosis process, and it has a negative impact on myocardial architecture and function, leading to cardiac remodeling, and associations with the severity of the coronary atherosclerosis considered in relation to the size of the ischemic area or the presence of HF [30,31]. Usually, higher levels of IL-6 are related to white race, female sex, impaired renal function (eGFR < 60 mL/min/1.73 m^2^), current smoking and severe coronary lesions [32]. Ridker et al. demonstrated that admission levels of IL-6 were predictive for future significant MACEs, such as non-fatal myocardial infarction, non-fatal stroke, cardiovascular death and MACE plus hospitalization for unstable angina requiring urgent revascularization [33,34]. In addition, compared to the lowest IL-6 levels, the highest levels were linked to a more than twofold greater risk for HF hospitalization and correlated with negative outcomes independently of recognized risk predictors and biomarkers (hs-CRP, highly sensitive troponin I (hs-TnI) and brain natriuretic peptide (BNP)). By stimulating the production of the matrix metalloproteinase (a protein family implicated in collagen production), IL-6 can increase fibrosis and cardiac remodeling, which are important aspects in the generation of HF over time [32].

#### 2.1.4. Interleukin-37

Interleukin-37 (IL-37) is an interleukin-1 (IL-1) family cytokine found in low levels in multiple locations, such as the brain, heart, colon, prostate and tonsils, and even in epithelial cells, keratinocytes, plasma cells, monocytes and macrophages [34]. Being an immunosuppressive factor, IL-37 plays a central role in both adaptive and innate immunity [35]. Kun et al. performed a case-control study in patients with ACS undergoing primary PCI. The study revealed that the baseline levels of IL-37 were, surprisingly, higher in the ACS patients (*p* < 0.05) compared to those with stable or without angina, especially in those with an electrically ischemic modification (*p* < 0.05), its levels being steadily elevated in relation to the degree of coronary artery disease (CAD) [36]. In addition, the same study [36] illustrated that reduced LVEF and raised N terminal pro-brain natriuretic peptide (NT-proBNP) levels correlated with increased levels of IL-37 before PCI, thereby independently anticipating in-hospital MACEs, acute HF, cardiac death, non-fatal myocardial infarction and target lesion revascularization (TLR). Although the anti-inflammatory mechanism of IL-37 in patients in ACS is yet to be definitively elucidated, Kun et al. proposed its role in the suppression of an acute and uncontrollable inflammatory process to be a feedback loop [36]. It has been anteriorly described that the expression of IL-37 is involved in the pathogenesis of atherosclerosis and that this biomarker has an inflammatory effect by reducing the expression of other inflammatory cytokines, such as interleukin-18 (IL-18) and TNF-α. Therefore, a stronger inflammation process may be associated with greater IL-37 levels. Moreover, higher levels of IL-37 expression on admission were positively associated with a greater mortality risk than lower levels [37].

#### 2.1.5. Procalcitonin

Procalcitonin (PCT) is an inactive precursor of calcitonin implicated in calcium homeostasis. Strong bacterial infections, major surgery and burns, various traumas and cardiogenic shock are some of the situations where serum PCT levels are greatly increased [22]. In recent years, PCT has received a lot of attention. It has been seen as a marker of the inflammatory response generated by AMIs. Dai et al., in a study that assessed the relation between myocardial infarction risk and plasma PCT levels, showed that an elevated concentration of PCT (immediate and average levels) was related to an increased risk of significant adverse cardiac events [38]. In contrast, other research has demonstrated the opposite effect. In a series of studies, PCT was recognized more as an infection predictor marker than a MACE post-ischemic factor. In a prospective trial, Barkey et al. concluded that PCT is more effective for ruling out infection, being a better biomarker than elevated CRP serum, white blood cell count and fever with a cut-off value of ≤0.09 ng/mL [39]. Reindl et al. measured the serum levels of PCT in patients with STEMI undergoing primary PCI within 24 h after pain onset (24 h and 48 h after the procedure) and compared them with cardiac magnetic resonance (CMR) markers for cardiac injury. The study found that serum PCT levels were not correlated with LV remodeling and negative clinical outcomes after an AMI [40]. Pavasini et al. [41] confirmed the role of PCT in predicting bacterial infection in patients hospitalized for ACS and cardiogenic shock (*p* < 0.001), even if its levels were greater in this group of patients despite not being associated with long-term outcomes or in-hospital mortality [42]. In addition, Hashemipour et al. assessed the association between the Gensini score as a measure of the severity and extent of CAD and PCT levels as a marker of inflammation in patients. They concluded that there was no statistical significance for the relationship between the two [43].

### 2.2. Biomarkers of Neurohormonal Activation

#### 2.2.1. B-Type Natriuretic Peptide

B-type natriuretic peptide (BNP) is a hormone produced as a consequence of ventricular dysfunction. Being secreted by cardiac cells, its roles include inhibition of the renin-angiotensin-aldosterone system, natriuresis and vasodilatation [44]. Recently, there has been interest in the deeper field of view; truncated molecular forms of BNP, such as BNP 5-32, BNP 4-32 and BNP 3-32, have appeared. Zubair et al. studied these types of molecules in 1078 patients with AMI. The focus was on the predictive capabilities of these molecules. The conclusion was that all of them were independently capable of predicting death, reinfarction, rehospitalization and MACEs, not only at 6 months, but also at 1 and 2 years after the main event. Therefore, they are associated with poor prognosis. In addition, BNP 5-32 was capable of improving upon the GRACE risk score for death at 6 months in combination with NT-proBNP. It could be considered a secondary risk stratification in these patients, particularly in low-risk form [45]. Carvalho et al. postulated that every modification that appears in BNP levels further leads to enhanced risk prediction, even in patients that do not experience associated HF in the course of an AMI. Furthermore, two measurements of BNP values, at days 1 and 5 after the event, were found to add a supplementary predictive aspect to the GRACE score system in comparison with using only a single measurement upon admission. Moreover, the same study reflected on the possible use of this biomarker for left ventricular degradation function at 30 days after STEMI when detected at significant levels. At the same time, it has been correlated with congestive HF, recurrent MI and mortality, regardless of short- or long-term follow-up [46]. Wolsk et al. demonstrated that, in patients with recent ACS associated with type 2 diabetes mellitus (which is known to interfere with values of BNP, regardless of the presence of a cardiovascular comorbidity), BNP along with NT-proBNP were great predictors not only for heart failure (*p* < 0.001) and death (*p* < 0.001) but also for stroke and myocardial infarction [47]. Moreover, in a study that included 180 patients with first-time AMI and primary PCI and follow-up at one year, Yan-Peng et al. examined the correlation between BNP and ST 2, IL-33 and MACEs. They observed that there was an important elevation in patients presenting with AMI with MACEs in comparison to those who did not present any MACEs. Moreover, there was a more diminished one-year survival rate. Another study observed a positive relationship between BNP, ST 2 and IL-33 and Gensini score; therefore, these markers were found to be assets for prediction of MACEs in a patient that had an AMI and underwent primary PCI [48]. Recently, Jun-Won et al. demonstrated that, in patients with AMI, in a prospective single-center PCI registry in which there was a measurement of BNP levels at admission and at follow-up of 2 months, short-term follow-up level after discharge was a stronger prognostic biomarker for MACEs and all-cause mortality [49]. Kazuhiro et al. described a particular situation: in patients with AMI and impaired glucose tolerance (IGT), high serum levels of BNP could predict the worsening of HF, the appearance of coronary stenosis and recurrent MI [50]. Furthermore, a fourfold increase in the risk of experiencing adverse cardiovascular outcomes at 10 months was observed in subjects with high BNP levels (>80 pg/mL) when compared with subjects who had consistently lower levels [51].

#### 2.2.2. Mid-regional Proadrenomedullin (MR-proADM)

Adrenomedullin (ADM), initially isolated from a pheochromocytoma tissue, is a ubiquitous peptide present in physiological conditions in vascular, renal, adipose, pulmonary and cardiac tissue. Increased plasma levels in patients with different diseases, such as congestive heart failure, hypertension, diabetes mellitus and myocardial infarction, were stimulated by volume overload [52,53]. Given its role in vasodilatation, natriuresis through inhibition of the aldosterone secretion, endothelial function and cardiac contractility, it is strongly correlated with the normal functioning of the cardiovascular system and is a good predictor of the risk for cardiac and non-cardiac death in heart failure patients [52,54]. Nonetheless, because of ADM’s short half-life in the bloodstream, its measurement is difficult. However, the assessment of mid-regional proadrenomedullin (MR-proADM), a precursor molecule of ADM with greater stability, is feasible [52,53]. A recent prospective study that evaluated the relationship between MR-proADM and the left ventricular function after an MI concluded that this biomarker is an independent positive predictor of adverse remodeling, one of the main causes of heart failure, and a negative marker of reverse remodeling [55]. The Danish Study of Optimal Acute Treatment of Patients With STEMI (DANAMI-3) found that high MR-proADM levels (≥0.79 nmol/L) prior to PCI in patients with STEMI were strongly associated with an elevated risk of short- and long-term all-cause or cardiovascular mortality and hospital admission for heart failure [56]. Furthermore, increased levels of mid-regional proadrenomedullin, measured within 24 h in patients with ACS and cardiogenic shock, were found to be a predictor of in-hospital mortality (*p* = 0.024) [54].

#### 2.2.3. N-Terminal Pro-B-Type Natriuretic Peptide (NT-proBNP)

N-terminal pro-B-type natriuretic peptide (NT-proBNP) is a prohormone released by cardiac cells in response to either myocardial stretching or an AMI. Despite the properties of BNP, NT-proBNP is associated with stability and a longer half-life time [57]. Moreover, natriuretic peptides have been proposed as a useful tool to indicate the degree of LV dysfunction in ischemic heart disease, arrythmias and cardiomyopathies, thus suggesting a need for a more intense approach to lower ventricular wall stress [58]. In a multivariable COX model study applied to an NSTEMI cohort, Gong et al. found NT-proBNP to be an independent risk factor for composite MACEs at 12 months after discharge, including all-cause mortality, hospital admission for unstable angina or heart failure, non-fatal recurrent myocardial infarction and target lesion revascularization (HR: 2.19, *p* = 0.0002). Furthermore, in patients older than 60 years old who were associated with an LVEF lower than 40%, this turned out to have a stronger prognostic value. Additionally, a positive correlation was found between NT-proBNP and the GRACE score (*r* = 0.58, *p* < 0,0001) [57]. In a retrospective study that included more than 200 patients with STEMI undergoing PCI, Zhao et al. studied the relationship between the presence of fragmented QRS (fQRS) in electrocardiograms in more than three leads or anterior leads and NT-proBNP as a prognostic marker of decreased regional ventricular systolic function [59]. The secondary analysis biomarker sub-study in the Platelet Inhibition in Patient Outcomes (PLATO) study found a substantial correlation between NT-proBNP levels and all-cause death after an ACS (HR: 2.96), as well as death secondary to HF (HR: 8.20), arrythmia (HR: 3.89) and sudden cardiac death. Therefore, it can be considered a predictive biomarker for all-cause and cause-specific mortality [60]. According to Tiller et al., in patients with STEMI undergoing primary PCI, the levels of NT-proBNP at admission were increased in those with an infarct size greater than 19% of the LV myocardium as evaluated by cardiac magnetic resonance imaging in comparison with those with a smaller affected area (140 ng/L vs. 86 ng/L, *p* = 0.008), an early assessment of the amount of the cardiac damage being an essential instrument for post-STEMI risk stratification [61]. Celebi et al. [62] showed that a single determination of NT-proBNP levels being greater than 400 pg/dL within 12 h offers a predictive value for LV aneurysm (LVA) development. A study by Zhang and Guo [63] revealed that high peak NT-proBNP levels were independently correlated with early onset LVA (OR: 1.08, *p* = 0.031), along with female sex, QS waves on electrocardiogram (ECG) and wall motion dysfunction.

#### 2.2.4. Copeptin

Copeptin is one of the biomarkers used in cardiovascular disease, as the C-terminal part of the pro-arginine vasopressin is secreted in equimolar quantities with it, and it also presents great stability in biological samples [64]. In the case of AMI, copeptin is known to show increased values immediately, after which the values decrease until the third to fifth days after the event, when it reaches a plateau phase. It has been shown that it may be a good prognostic factor for STEMI, as well as for HF or ischemic stroke [65]. Lattuca et al. demonstrated, in an unselected cohort of 401 STEMI patients, that values of copeptin determined at admission presented an independent and feasible prognostic marker for one-year mortality (*p* = 0.022), being even better than the peak cardiac troponin I levels studied under the same conditions. Copeptin could encourage more aggressive treatment [66]. Furthermore, Ahmed et al. also proved the greater value of copeptin compared to troponin I for NSTEMI patients and its prognostic value for MACEs (cardiac death, re-infarction, re-hospitalization for ischemic events, HF, stroke and TLR) and coronary revascularization within one year of follow-up (*p* < 0.001 for each) [67]. In a study conducted by Marta et al. [64] that included 100 patients with AMI undergoing primary PCI, increased copeptin levels on the fourth/fifth day, but not on admission, were associated with MACEs (re-infarction, unscheduled coronary revascularization and all-cause death) (*p* = 0.024). Recently, copeptin was found to be an independent predictive factor for total mortality but also a marker for general vulnerability, being influenced by the presence of HF, diabetes mellitus type 2, female sex and previous MI [68]. In previous research that assessed the relationship between copeptin and LV systolic function using a two-dimensional global longitudinal strain (GLS) in STEMI patients, based on the median copeptin level, it was found that there was a negative correlation between its values and early or 6 month GLS (*r* = −0.45 at the early stage; *r* = −0.662 at 6 months) [69]. In addition, according to Ersin and Ayca, it was found that onset copeptin levels were an independent predictor for contrast-induced nephropathy in patients with STEMI treated by PCI [64].

### 2.3. Biomarkers of Myocardial Necrosis

#### 2.3.1. Platelet-Related Biomarkers

Platelets (PLTs) are small and anucleated cells that are derived from megakaryocytes secondarily to a maturation process that takes place in bone morrow. They are then re-leased into the bloodstream, where they have a limited lifespan of 5–7 days. They are multifunctional cells that play an essential role in primary hemostasis and thrombosis, addressing any hemorrhagic situations that appear at the level of the vascular endothelium or tissue as the result of an injury [70,71]. Furthermore, in the case of AMI, there tends to be a paradigm in which PLT can actually contribute to the formation of occlusive thrombosis as a consequence of plaque disruption [71]. Mean platelet volume (MPV) is an indirect, helpful, reliable and detectable indicator of platelet activity. Many studies demonstrate the correlation between MPV and MACEs in patients with ACS [72,73]. In retrospective research conducted by Avci et al. [74], the authors proved that an increased in-hospital MPV in STEMI patients undergoing PCI is an independent predictor factor of all-cause mortality (HR: 1.301, *p* = 0.008), MPV being measured upon admission and 2–3 days after. In a study that enrolled 1094 patients with ACS, Chang et al. [75] found that MPV was considerably greater in those with myocardial infarction than in the non-ACS group (8.6 ± 1.1 vs. 8.4 ± 1.0 fl, *p* = 0.002), high levels of MPV being associated with MACEs (all-cause mortality, recurrent ACS, target vessel reintervention and stroke). In particular, a retrospective study showed that high levels of MPV (MPV ≥ 9.0 fl) in patients with NSTEMI were associated with a substantial CAD (*p* = 0.005), defined as ≥ 1 major coronary artery stenosis (≥70%) [76]. In contrast with other researches, Çanga et al. [77] found no correlation between CRP and short-term prognosis in young patients with STEMI; only MPV (≥9.8 fl) was able to predict cardiovascular death and non-fatal reinfarction within 30 days upon admission (*p* < 0.01). Furthermore, greater mean platelet volume was identified as a predictor of death in patients with AMI throughout their hospitalization [78]. The primary objective of AMI therapy is to promptly revascularize the obstructed arteries by either fibrinolysis or pPCI, with the no-reflow phenomena reducing the benefit of the second method and predicting negative outcomes. Hence, Kurtul and Acikgoz [79] proved the positive correlation between high MPV levels and no-reflow phenomena (MPV ≥ 8.65 fl). They illustrated that a cut-off value greater than 4.87 fl is a predictor of in-hospital mortality in comparison to those with lower cut-off levels (7.3% vs. 2.6%, *p* < 0.001). Recently, a meta-analysis that grouped 21 studies concluded that a higher MPV is associated with reduced post-intervention coronary flow (OR: 2.13, *p* < 0.0001) [80]. Moreover, in patients with STEMI treated with PCI, the MPV to platelet count (MPV/PC) ratio proved to be a predictor for long-term mortality and non-fatal reinfarction and, thus, a better prognostic marker than MPV alone [81,82].

#### 2.3.2. Troponins

Troponins (T, I, C) (cTnT, cTnI, cTnC) are structure proteins that are implicated in actin–myosin interaction. They are produced and released from cardiomyocytes in response to increased stretch or necrosis. Hence, cTnT and cTnI, which are known as cardiac-specific troponins, have been proven to have and important role as a diagnostic marker for cardiac injury and AMI [31,83]. After an ACS, their levels rose within 2–4 h and reached a peak at 24 h, with a persistent elevated value for 2–3 weeks [84]. In ACS, time is precious and thus there is a need for a quick diagnostic tool to help guide treatment, such as these highly sensitive troponins (hs-cTnI and hs-cTnT). Zeljković et al., in a prospective study, presented mean values of cTnT (*p* = 0.01) together with peak levels of creatine kinase (CK) (*p* = 0.0008) as a long-term predictor of impaired LV systolic function < 50% in STEMI patients with preserved systolic function at onset and single-vessel coronary disease [85]. Along these lines, Mohammad et al. demonstrated in recent research on 578 STEMI patients undergoing PCI that hs-cTnT values were positively correlated with long-term LV systolic dysfunction (defined as LVEF ≤ 40% after one year), thus suggesting that this biomarker can be a useful instrument in identifying those patients at high risk of adverse cardiac remodeling [86]. However, the RUTI-STEMI real-life cohort study has not proven the prognostic role of new generations of troponins in STEMI [87]. Wanamaker et al. demonstrated that in-hospital mortality is increased in patients with high troponin upon admission, regardless of other clinical risk factors [88,89]. In a retrospective study of 818 patients with STEMI undergoing pPCI during the first day of the symptom’s onset, both admission (HR: 1.08; *p* < 0.001) and peak post-procedural hs-cTnT levels (HR: 1.06; *p* < 0.001) were independently correlated with all-cause mortality over up to 3 years of follow-up [90]. Additionally, Harada et al. [91] has proven that greater post-procedural levels of hs-cTnT in patients with NSTEMI treated with early PCI are related to an increased risk of all-cause and cardiac death up to one year after the acute event. In contrast, there are data suggesting that high levels of cTnI post-pPCI do not predict three-year cardiovascular and all-cause mortality [60,92]. In addition, a recent meta-analysis concluded that raised cTn levels are an independent predictor factor for all-cause mortality in patients with ACS [93,94].

#### 2.3.3. Creatin Kinase-MB (CK-MB)

Creatine kinase-MB (CK-MB), an isoenzyme of enzyme CK located mostly in cardiac cells, and in low levels in skeletal muscle, is released into circulation within 4–6 h after a cardiac injury, reaching a peak at 24 h and returning to normal after 48–72 h. In a Dutch study that enrolled 1360 STEMI subjects with a median follow-up period of 6.7 years, heart failure occurred in 85 patients (6.3%). Peak creatinine-kinase values, together with a left anterior descending artery culprit lesion, have been found to be an important predictor of heart failure development [95]. In the same line, Yang et al. and Hendriks et al. proved the utility of an early measurement of CK-MB levels in predicting left ventricular adverse remodeling after an acute myocardial infarction and, thus, heart failure onset [96,97]. A previous study that included more than 2000 NSTEMI patients treated with early PCI [98] revealed that peak post-procedural CK-MB (greater than three times the ULN cut-off) is an independent predictor of three-year mortality compared to lower levels of CK-MB. Other studies have shown that, in subjects with STEMI who underwent PCI, the CK-MB serum levels correlated with in-hospital mortality [99,100]. Furthermore, in a multivariate analysis, the incidence of contrast-induced nephropathy was substantially higher in those with increased CK-MB values when compared with the non-CIN group (*p* = 0.001) [101]. In addition, Hsu et al. [51] proved that, in patients with AMI, early peak CK-MB levels (cut-off 48.2 ng/mL) were associated with an increased risk for adverse cardiac remodeling within six months after discharge. Additionally, patients with log-transformed CK-MB values greater than 4.7 had a 3.4-fold higher incidence of CIN than those without [102]. Peak CK-MB showed a strong association with chronic scar dimension and chronic wall motion abnormality index in patients with non-transmural myocardial infarction undergoing PCI or coronary artery bypass graft surgery (CABG) [103].

#### 2.3.4. Cystatin C (cysC)

Cystatin C (cysC) is a pleiotropic cysteine protease inhibitor that regulates cathepsins S and K in human vascular pathology, being a part of the catabolism processes of most proteins in nucleated cells. In normal conditions, cysC is an indicator of renal function, with greater sensitivity than serum creatinine for incipient stages of renal function. It could also be present in cardiomyocytes, showing an important increase in a hypoxic scenario [104,105,106]. It is also known that cysC is part of processes such as aging, apoptosis, destruction of proteins in the extracellular matrix, cell differentiation and proliferation and synthesis of oxide nitric (NO) [107]. In AMI, the prognostic significance of cysC may be attributable to multiple underlying mechanisms. First, it has been suggested that cysC plays an active role in the physiopathological process of atherosclerosis plaque formation. Furthermore, being an endogenous cathepsin inhibitor, it maintains the balance between proteases and their principal inhibitor. An increased level indicates the disruption of this relationship, therefore accelerating atherosclerosis development [108]. Secondly, elevated cysC values may lead to no-reflow phenomena due to its correlation with impaired renal function and inflammation, which are associated with oxidative stress, microvascular endothelial dysfunction, pro-coagulant cytokines and free radicals. Therefore, this biomarker is a useful predictor for no-reflow events in STEMI patients treated with pPCI [109]. Recently, Lou et al. [110] presented cysC levels at admission as a biomarker of cardiac function, and they showed a negative relationship with the ejection fraction value (*p* < 0.0001) and functioned as a predictor for MACEs, cardiovascular mortality and all-cause mortality in AMI patients within 4 years of follow-up. In addition, elevated levels of cysC measured at any stage within the first year after an ACS positively correlated with the composite endpoint (cardiac mortality, non-fatal AMI and unplanned coronary revascularization) (*p* = 0.006). Further, the 12th to 14th day measurements demonstrate an important predictive role regarding adverse cardiovascular outcomes within 3 years of follow-up [111,112]. On the same note, a meta-analysis conducted by Chen et al. [105] suggested that increased serum cysC levels positively correlated with a higher risk of MACEs and mortality in patients with STEMI undergoing PCI. Similarly, there are data that present cysC as a predictor of negative cardiovascular events, such as cardiovascular death, hospitalization for HF, recurrent cardiovascular events and the severity of vascular lesions, with cysC being strongly correlated with the Gensini score (*p* < 0.05) in acute coronary syndrome patients [113,114,115,116]. Even if the retrospective study conducted by Chen et al. [117] included subjects with elevated cysC levels who were older and had hypertension, advanced Killip classes and a reduced eGFR, after adjustment for these confounders, this biomarker was found to be an independent predictor for negative cardiovascular outcomes and death in STEMI patients treated with late PCI (more than 7 days from symptom onset). In contrast with the aforementioned study, a sub-analysis of the HIJ-PROPER demonstrated that levels of cysC greater than 1.03 mg/L are independent predictors only for all-cause mortality but not for cardiovascular events [118]. In addition, in a cohort of patients with STEMI undergoing pPCI, plasmatic cysC did not predict acute kidney injury, one-year mortality or hemodialysis necessity after one year [119].

#### 2.3.5. Heart-Type Fatty Acid Binding Protein (H-FABP)

Heart-type fatty acid binding protein (H-FABP) is a cytoplasmatic protein involved in the metabolism of fatty acid in the myocardium. Its level increases promptly (< one hour) during a myocardial injury, with a peak at 4–6 h and return to baseline after 24 h [120]. Therefore, as it is detectable in plasma before cTn, it can be considered an early biomarker of AMI and a useful tool in reinfarction prediction [121,122]. The association between H-FABP and stable coronary heart disease has been reported in several studies as an independent predictor for cardiovascular events and acute HF-related hospitalization. A group of patients with high H-FABP levels had a 1.5-fold higher rate of negative outcomes than the lowest-level group [123,124]. Even if H-FABP is quickly released into the bloodstream and is a stable and soluble heart tissue-specific protein with high concentration in myocardium, there are no recent data regarding its prognostic value [120]. However, there are some reviews in which H-FABP seemed to have some prognostic value for patients with acute coronary syndrome, but supplementary studies are needed to evaluate whether there is a correlation between this and adverse outcomes [125,126].

#### 2.3.6. Endothelial Cell-Related Biomarkers

The endothelial cell-specific molecule-1 (ESM-1), or endocan, is a soluble proteoglycan produced by human vascular endothelial cells. It is implicated in numerous processes, such as cell adhesion, migration, neovascularization and proliferation. Its secretion is upregulated by a series of proinflammatory cytokines (TNF-α, interleukin-1β and angiogenic factors, such as vascular endothelial growth factor), suggesting that it plays a key role in inflammation and endothelial dysfunction in various vascular disorders (angiogenesis, atherosclerosis, infections and neoplasms) [127,128,129]. It is known that endocan is involved in the pathogenesis of cardiovascular disease, higher levels of it being observed in subjects with coronary slow-flow, hypertension and coronary artery disease [130]. Ziaee et al. [131] claimed that elevated endocan levels at admission in patients with ACS were a predictor of MACEs (OR: 3.231, *p* < 0.001), defined as HF, recurrent ischemia or in-hospital death, at hospital admission or at 30-days follow-up, being positively correlated with the thrombolysis in myocardial infarction (TIMI) risk score. A recent study has postulated that endothelial dysfunction and inflammation mediated by high concentrations of endocan could generate poor cardiomyocyte microvascular perfusion and, thus, create incomplete ST-segment resolution (STR) and extensive myocardial damage [132]. Cimen et al. [133] followed 35 patients with ACS treated with CABG and measured the endocan levels before and after the procedure, concluding that endocan can be a useful instrument in predicting a successful reperfusion. A recent meta-analysis found that endocan may be positively associated with MACEs, coronary artery disease mortality and disorder severity [130]. Dogdus et al. [134] enrolled 137 STEMI patients treated with pPCI and reported that endocan value is an independent predictor of no-reflow phenomena and of a worse clinical outcome.

#### 2.3.7. Aspartate Transaminase

Aspartate transaminase (AST) is a liver transaminase and is especially derived from its levels at this location. It can also be found in other locations, such as the heart, muscles and red blood cells. Therefore, this biomarker presents an imperfection with regard to its liver function specificity [135]. Data have shown that cardiovascular diseases are often accompanied by impaired liver function (as a result of reduced arterial perfusion or congestion); hence, this transaminase’s activity as a prognostic factor for morbidity and mortality in cardiovascular patient groups has been the main focus of studies. In a retrospective center study conducted on 569 AMI patients, AST was found to be a predictor factor for cardiac death and MACEs (AMI, unstable angina, TLR or another segment or coronary vessel, stroke and saphenous vein aortocoronary bypass grafting) at 6 years of follow-up, its value being significantly correlated with troponin and myoglobin [136]. Li et al. [137] reported that, in AMI patients, increased AST was not an independent predictor for in-hospital mortality, in contrast to alanine aminotransferase (ALT), which was found to be a marker of elevated risk of early death, together with older age; increased d-dimers, fibrinogen or fasting plasma glucose; and decreased eGFR. A prospective study that assessed the association between AST levels and all-cause mortality in STEMI patients determined a predictive value for levels greater than 492 U/L for all-cause mortality at short- and long-term follow-up, with these levels being correlated with Killip classification, pre-TIMI flow, cTnI and infarct-related coronary artery [135]. A series of studies found that the ratio of AST and ALT, known as the De-Ritis ratio, is an independent predictive factor for long-term mortality (*p* < 0.001) in AMI patients. A value greater than 2 for this ratio is an indicator of total occlusion of the culprit lesion. Furthermore, the De-Ritis ratio has predictive value in association with NT-proBNP, troponin T and CK [138,139].

#### 2.3.8. Other Biomarkers of Myocardial Necrosis

Myoglobin, a hemoprotein expressed in the cytoplasm of striated muscle cells (cardiac and skeletal muscle), has long been used as a biomarker in the early detection of acute coronary syndrome due to its rapid release into circulation (within the first 30 min). Unfortunately, due to its limited cardiac selectivity, attention has shifted to troponins [84,140]. Furthermore, even if studies 20 years ago identified myoglobin as a predictive factor for short- or long-term death, there are little data from the previous five years to indicate its efficacy for prognoses in patients with ACS [141,142]. There is one study that found that when the log (myoglobin) transformation increased by one unit, the risk of mortality increased by 6.9 times [143].

Cardiac myosin-binding protein C (cMyC) is a structural cardiac protein that has fivefold greater cytoplasmic concentrations than cardiac troponins. It peaks in plasma early in the event of an ACS, showing the same diagnostic performance as cTn [144,145]. According to Kaier et al. [146], risk prediction appears to be quite similar when comparing hs-cTn and cMyC. A level lower than 10 ng/L has a significant negative predictive value and indicates a 30 day mortality rate close to zero, with the capacity to rule out an AMI within 2 h of onset of symptoms [147].

Ischemic-modified albumin (IMA) is a type of human serum albumin. It is very sensitive and identifiable in the early reversible stage of ACS, in which the N-terminal amino acids have been altered by pathological conditions, such as hypoxia, acidosis, free radical damage and energy-dependent membrane disruption [148]. Ding and Yang [149] demonstrated that the rate of MACEs was higher in patients with elevated levels of IMA in contrast to those with decreased levels within 1 year of follow-up after an AMI. In a previous study, IMA was found to be a predictor of the severity of coronary artery disease. Higher levels were related to greater risk of CAD but they did not correlate with the Gensini score [150].

Lactate dehydrogenase (LDH) is an enzyme that catalyzes the conversion of lactate to pyruvate and back the conversion of NAD+ to NADH and back. According to a recent prospective study that looked at the link between LDH and left ventricular function after MI, this biomarker is an independent positive predictor of unfavorable remodeling, which is a leading cause of heart failure, and a negative marker of reversal remodeling [51].

### 2.4. Cardiac Remodeling Biomarkers

#### 2.4.1. Galectin-3

Galectin-3 (Gal3), part of the galectin family, is a glycoprotein that binds at a variety of locations where it can be found, such as the spleen, stomach, heart, liver, pancreas and uterus, as well as being expressed by all type of immune cells, epithelial cells and endothelial cells [22,151,152]. It plays a critical role in various biological processes, such as cell proliferation, angiogenesis, apoptosis, differentiation, fibrosis and immunity [151]. By promoting phagocytosis, Gal3 reverses the switch from inducible nitric oxide synthase to arginase in plaques, both of which are important in atherogenesis [22]. Furthermore, this molecule plays a role in amplifying the inflammatory response, particularly in the vascular wall, by stimulating the macrophage secretion of inflammatory cytokines. Then, the macrophages turn into foam cells, which is an important stage in the atherogenesis process [153]. Several studies have pointed out that Gal3 is strongly associated with poor outcomes, such as new onset atrial fibrillation (demonstrated at levels greater than 16 ng/mL), left ventricle (LV) remodeling, heart failure and all-cause mortality [152,154,155,156]. In addition, Gal3 was an independent predictor for LV remodeling at 4 months of follow-up. Therefore, Gal3 remains an important clinical biomarker in predicting adverse outcomes in patients with ACS, such as cardiovascular death, all-cause mortality and heart failure. It can be used as a target treatment for preventing or reversing heart remodeling [152]. It was observed that its levels are reduced following administration of mineralocorticoid receptor antagonists. Furthermore, Branka et al. observed that treatment with trimetazidine and long-acting nitrates reduced the levels of Gal3 within 30 days in both NSTEMI and STEMI patients [152,157]. The expression of Gal3 in infarcted areas follows a dynamic pattern, strongly correlated with the presence of other biomarkers implied in myocardial remodeling [158]. Usually, Gal3 presents low expression in healthy myocytes but, when cardiac damage appears, such as in an AMI, its levels rapidly grow, thus contributing to the early stages of tissue healing. As a result, Gal3 plays a critical role in the initiation and progression of restoration processes in the affected area [132]. Ben Ahmed et al. [159] proved that the number of patients with STEMI who showed LV dysfunction was significantly higher among those who presented increased levels of Gal3 at admission (*p* = 0.003). Additionally, they proved that there is a positive correlation between the number of vessels affected and Gal3 levels (*p* = 0.04) [153]. There are multiple studies that illustrate Gal3 to be an independent predictor marker for lower LV function and negative cardiovascular outcomes. Recently, Asley et al. suggested a positive correlation between Gal3 levels and mortality and heart failure post-AMI [155,158,159]. Measuring plasmatic levels of Gal3 on day 30 after an AMI functioned as an independent risk predictor for both diastolic and systolic dysfunction within 6 months of a post-ischemic event. This correlation is more pertinent for prognosis than levels measured on day 1 or 5 [157,160]. In contrast, Di Tano et al. [154] demonstrated the usefulness of evaluating Gal3 plasma levels immediately post-AMI following PCI for the prediction of the onset of LV remodulation in patients hospitalized with a PCI-treated anterior STEMI. Furthermore, galactin-3 can be considered a new biomarker that can predict the complexity of coronary lesions on its own, being independently associated with the SYNTAX score and electrocardiographic measures of ST segment resolution in patients with STEMI undergoing primary percutaneous intervention (pPCI) [161]. The role of Gal3 in anticipating the risk of death and heart failure with increasing levels of Gal3 was also proven by Asleh et al. [155] but, in addition, they suggested that its prognostic value is independent of myocardial infarction severity, comorbidities, levels of troponin T or soluble suppression of tumorigenicity-2 (sST2). Further, Gal3 may be related to the onset of atrial fibrillation; its levels are greater in patients with AMI and atrial fibrillation (AF) than those that do not show this type of arrythmia. Therefore, dilated left atrium (LA) and ventricle, as well as an increased risk of AF (paroxysmal or persistent/permanent), were both noted in those with high concentration of this biomarker. A level greater than 7.57 ng/mL was associated with increased risk of AF, suggesting possible worse outcomes. Therefore, there is a need for more intensive treatment in these patients [162]. In addition, Przemyslaw et al. found, in a pilot study undertaken on patients with AMI, that Gal3 levels greater than 9.2 ng/mL at discharge alone correlated with a ninefold increase in the risk of composite endpoint appearance [163]. George et al. and Przemyslaw et al. showed that there is a significant negative association when it comes to estimated glomerular filtration rate (eGFR) and levels of Gal3, low rates being observed in those presenting high concentrations of Gal3; at the same time, it was shown that there was a positive concordance with creatinine levels (*p* = 0.02) [159,163]. In the study performed by Agatha et al., Gal3 presented increased baseline levels in patients with STEMI who reached the primary endpoint (HF occurrence at 1 year follow-up) (*p* < 0.001) [164]. In another study undertaken on patients with first-time STEMI treated with primary PCI, Gal3 was an independent predictor for cardiovascular disease or hospitalization for HF in 1 year of follow-up. In addition, a high concentration of this biomarker was associated with an increased Killip class at admission or with a need for intensive diuretic treatment during hospitalization, as well as with higher risks in GRACE and TIMI scores [165].

#### 2.4.2. Soluble Suppression of Tumorigenicity 2 (sST2)

Soluble suppression of tumorigenicity 2 (sST2) is an isoform-type of the protein ST2. It is implicated in the homeostasis and pathogenesis of multiple inflammatory and cardiac pathologies and autoimmune diseases. It is especially expressed in those processes that imply fibrosis, remodeling or tissue lesions [166]. Therefore, sST2 may be seen as a unique pathway in prognostic outcomes after an AMI. In a prospective study that included 380 patients with STEMI grouped in two categories (low vs. high sST2 serum concentrations), Somuncu et al. found that, after 12 months follow-up, mortality was statistically significant higher in the elevated sST2 group (15.5% vs. 4.9%, *p* = 0.001). Apart from this, notable MACE values (HF, stroke, cardiovascular mortality, non-fatal reinfarction and target vessel revascularization) were greater in the same group [167]. Other investigators have postulated that the risk of adverse cardiac outcomes during intensive care for STEMI was about six times greater in patients with sST2 supramedian concentrations on admission (with a median level of sST2 = 246.6 ng/mL compared to STEMI without adverse cardiac events = 122.9 ng/mL, measured in point-of-care testing (POCT) format). They defined MACEs as a composite of cardiac death, acute heart failure, resuscitated ventricular arrhythmia (ventricular tachycardia/ventricular fibrillation), cardiogenic shock and reinfarction. Hence, sST2 POCT assessment can be a valuable asset to independently predict MACEs during acute intensive care for STEMI [168]. Aleksova et al. [169] measured circulating levels of sST2 in patients with STEMI types 1 and 2 and proved that values greater than 70 ng/mL positively correlated with a major activation onset in fibrotic and neurohormonal directions and, thus, adverse reverse LV remodeling, suggesting a need for an aggressive therapeutic approach in this case (with spironolactone or the combination sacubitril/valsartan). A study with a median follow-up of only 180 days undertaken on patients with diabetes and ACS revealed that high levels of sST2 remained an independent predictor of MACEs (unstable angina/NSTEMI, recurrent AMI, stroke, rehospitalization and death) in the short term [170]. Although the values of sST2 tend to be higher in patients with AMI, studies have illustrated that the higher the values, the greater the risk of death and HF, regardless of the presence of other clinical prognostic markers. In a prospective longitudinal cohort of patients showing incidence of AMI, Jenkins et al. found that, after 5 years of follow-up, those with levels of sST2 greater than 72.3 ng/mL had mortality that was 52% greater compared to those with low levels of sST2. Therefore, there was a sixfold increase in the risk of death (*p* < 0.001) [171]. In a prospective, non-randomized, single-center study, Zagidullin et al. demonstrated that, in patients with STEMI, sST2 had a better prognostic value for cardiovascular (CV) mortality at two years in association with NT-proBNP and pentraxin-3 (Ptx-3), rather than alone, or in combination with only one other value [172]. Additionally, a recently meta-analysis that aimed to evaluate the diagnostic value of sST2 in CV diseases suggested that its prognostic value is often a negative one, and it can predict the LV function after AMI; however, further investigations are required [173]. A series of studies assessed the cut-off value of sST2 in patients with STEMI undergoing primary PCI for a variety of MACEs at 1 year follow-up (such as impaired myocardial revascularization, HF, all-cause death, non-fatal myocardial infarction and non-fatal stroke), finding mean variation levels greater than 75.8 ng/mL, 58.3 ng/mL or even 2.003 ng/mL (for the revascularization part) [173,174,175,176].

#### 2.4.3. Growth Differentiation Factor-15 (GDF-15)

The transforming growth factor-beta (TGF-beta) family of proteins includes a subfamily of proteins known as growth differentiation factors (GDFs). GDF-15 is increased in response to cardiac damage and the inflammatory process, thereby increasing the risk of an elevated cardiovascular event [177]. In the presence of an ACS, a single measurement of GDF-15 and concentrations greater than 1800 ng/L were linked to an elevated risk of all-cause mortality, MACEs and hospitalization for heart failure, but not subsequent readmission for MI [178,179]. Additionally, GDF-15 concentrations were assessed at baseline in 4330 patients, and the same cut-off value was linked to a twofold increase in the risk of CV death/HF [180]. Furthermore, a recent study found that elevated GDF-15 levels (>2350 pg/mL) detected in the first 24 h following the onset of ACS were independently linked with an unfavorable prognosis within 5 years of the event [181]. The stratification of the risk of these individuals using cumulative doses of TnT/NT-proBNP and GDF-15 has proven to be highly effective [182].

#### 2.4.4. Syndecan-1 (Sdc1)

Syndecan-1 (Sdc1) is a surface protein that is a component of the endothelial glycocalyx (eGC), a complex of transmembrane proteins that are found on the endothelial surface of blood vessels. Damaged eGC-soluble sdc1 that can be used as an indicator is produced during shedding. An Sdc1 level greater than 148 ng/mL was 14 times more likely to lead to an ACS diagnosis (95% CI: 1.8 to 102) [183]. In terms of its function in predicting subsequent cardiovascular events in acute reperfused STEMI patients, Sdc1 > 120 ng/mL was independently linked with death at 6 months [184]. Furthermore, syndecan-1, in association with CRP and TnT, can act as an independent predictor for microvascular occlusion (MVO), a condition characterized by “no-reflow” phenomena in patients with STEMI following pPCI [185].

#### 2.4.5. Circulating LIPCAR

LIPCAR, a long noncoding RNA (lncRNA), is located in the nucleus and cytoplasm. It lacks the capacity to code for proteins, but it may still control gene expression through epigenetics and may serve a variety of purposes inside the cell. According to a cross-sectional study at Shanghai Gong Hospital’s Department of Cardiology, LIPCAR levels were higher in HF patients following an AMI than in patients without HF, and the test had a high positive predictive value for the diagnosis of HF. The findings suggest that LIPCAR may be a biomarker of early HF following AMI [186]. In patients with STEMI, greater levels of LIPCAR were found to be independent predictors of significant adverse cardiovascular events (HR = 5.93; 95% CI, 1.46–9.77). Furthermore, a correlation study showed that LIPCAR was negatively linked with LV ejection fraction and favorably correlated with myocardial enzymes. However, further research is required to fully understand LIPCAR’s therapeutic application value and expression process [187].

#### 2.4.6. Thrombospondin-1 (TSP-1)

Five atrial extracellular matrix (ECM) member proteins form the thrombospondin family, with thrombospondin-1 (TSP-1) being the best known due to its role in inflammation and fibrogenesis in several illnesses, including liver fibrosis, diabetes, various cancers and cardiovascular conditions [188]. TSP-1, which controls inflammatory responses via the transforming growth factor-1 (TGF-1) pathway, is mostly released by macrophages and platelets [189,190]. A total of 24 individuals out of a total of 219 patients with AMI who underwent PCI and had no prior arrhythmias developed atrial arrhythmias (AAs). TSP-1 levels were greater in AA patients than in non-AA patients. As a result, thrombospondin-1 has become an independent risk factor for AA in patients with AMI [191].

#### 2.4.7. Uric Acid

Uric acid (UA) is the final degradation product of purine nucleotides from endogenous (cellular nucleoproteins) and exogenous origins (alimentary) [192]. In the Framingham study that included 5127 participants followed up for 12 years, Kannel et al. established the link between UA and CV events by demonstrating an elevated risk of myocardial infarction in people with hyperuricemia [193]. Since then, several further studies have supported this relationship, and hyperuricemia has gained recognition as a distinct CV risk factor. The independent relationship between increased UA and long-term mortality and MACEs in ACS patients following PCI has been documented in recent research, despite years of controversy around the use of UA as an independent prognostic marker in patients with ACS [194]. Prior investigations revealed an independent relationship with UA in patients presenting with ACS with regard to short-term prognosis [195,196], with peak rather than admission UA levels predicting both 30 day and 1 year mortality [197]. Data on the relationship between UA and long-term outcomes are limited. However, hyperuricemia has been linked to an increased risk of 2 and 5 year all-cause mortality in STEMI patients following PCI [198,199], with the best cut-off value for UA to predict MACEs in young patients with NSTEMI being >5.2 [200]. Specifically, a number of studies discovered a connection between UA levels at admission and particular HF-related problems, such as the Killip class, the use of an intra-aortic balloon pump and cardiogenic shock, LV ejection fraction at admission, and the use of non-invasive ventilation [201,202]. Furthermore, with regard to STEMI patients, the plasma level of uric acid at admission continues to be a good indicator of impaired coronary blood flow after the first PCI [203]. A meta-analysis on this subject found that, when compared to patients with low UA, STEMI patients with high UA had a higher incidence rate of in-hospital MACEs, in-hospital death and one-year mortality [204]. Nevertheless, despite substantial studies, the potential association between hyperuricemia and cardiovascular disease is still debatable; more research should be conducted to better understand how this component fits into the CV pathology spectrum and whether it might serve as a target for CV preventative measures.

### 2.5. Vasoactive Biomarkers

#### 2.5.1. Neuropeptide-Y

A powerful vasoconstrictor with adverse cardiac inotropic and chronotropic effects, neuropeptide Y (NPY) is a peptide co-localized and co-released with NA from sympathetic nerve terminals, particularly during situations of sympathetic hyperactivity, such as myocardial infarction [205,206]. According to research, NPY is the most prevalent neuropeptide in the heart and is dramatically enhanced during pPCI for STEMI, its level being elevated for at least 48 h after revascularization [205]. Prior to the invention of pPCI, clinical trials showed that higher peripheral levels of “NPY-like activity” following myocardial infarction were associated with a higher risk of death at 1 year [206]. A previous study found that infusing NPY into coronary arteries in humans caused ischemia ECG alterations and chest discomfort. This effect may be due to NPY’s ability to selectively constrict the coronary microvasculature via the Y1 receptor [207,208]. The first goal in patients with STEMI is to revascularize the infarct-related epicardial artery as soon as possible by utilizing primary percutaneous coronary intervention. Despite this, one third of patients experience “no-reflow”, a condition in which there is insufficient myocardial reperfusion. This entails a persistent restriction of the flow of blood via microcirculation and is linked to prolonged ST-segment elevation, a wider infarct area, a lower left ventricular ejection fraction, frequent heart failure admissions and overall mortality [209]. The underlying cause of this is as-yet unknown but is most likely complex, and the symptoms of myocardial ischemia/reperfusion injury to the myocardial microcirculation vary from transient edema to microvascular damage with intramyocardial bleeding [210]. At the time of the pPCI, neuropeptide-Y levels analyzed from a peripheral vein or coronary sinus were independently associated with coronary microvascular dysfunction, increased cardiac injury, decreased LV ejection fraction 6 months after the acute event and subsequent heart failure and mortality over an average follow-up of 6.4 years [211]. Additionally, when used in experimental ST-elevation ischemia and reperfusion, NPY (250 nmol/L) dramatically enhanced the incidence of VT/VF (60% vs. 10%), and this could similarly be avoided by the Y1 receptor antagonist BIBO3304. Drugs that block the Y1 receptor enhance the beta-anti-arrhythmic blockade’s effects and may provide a distinct pharmacological approach in patients with ventricular arrhythmias [212]. Furthermore, inhibiting NPY Y1 receptors may make it possible to attenuate the consequences of no-reflow after STEMI, which resulted in better survival in a subgroup of subjects with elevated initial NPY [211].

#### 2.5.2. Neuregulin-1

A member of the epidermal growth factor family, neuregulin-1 (NRG-1) is crucial for the development of cardiomyocytes and cardiac regeneration. There are six isoforms, and each one has an EGF-like domain that is both required and sufficient to activate ErbB receptors and, thus, exerts essential biological activities [213]. Prior studies have shown the following: NRG1 undertakes anti-fibrotic and anti-remodeling actions in heart failure models, it protects myocardial cells from damage and it improves cardiac function [214]. NRG-1 has been shown to have an anti-inflammatory impact on activated monocytes through a mechanism that modulates ErbB3 signaling, which causes a noticeable decrease in the production of pro-inflammatory cytokines [215]. Accordingly, a study found that reperfused myocardial infarction in rats was enhanced by the tissue-protective technique known as remote ischemic conditioning (RIC), which stimulates NRG-1 production in the infarcted tissue location [216]. Although the use of human-recombinant NRG-1 in HF patients significantly increased cardiac output and LVEF, its circulating expression and functional significance as a biomarker in the development of unfavorable post-MI remodeling in patients with STEMI are still unknown [217]. Research conducted by Haller et al. showed that circulating NRG-1 levels in STEMI patients decreased following PCI, with no correlation with other variables, such as total ischemic time, RIC and LV ejection fraction [217,218]. On the other hand, individuals at advanced stages of HF were identified by a large increase in circulating NRG-1 levels but with a reduction in the mRNA expression of ErbB2 and ErB4 [218,219]. Despite being efficacious in rats, no effect from RIC on plasma NRG-1 levels was found [216,218]. Even if some studies have suggested a beneficial effect on myocardial recovery in the context of STEMI, the overall benefit of RIC appears to be limited [220]. Additionally, the decline in NRG-1 may serve as a marker and driver for the emergence of microvascular obstructions and diastolic dysfunction following MI. In accordance with this, a recent report demonstrated that a catheter-based intramyocardial injection of NRG1-loaded microparticles significantly improved left ventricular diastolic function in a preclinical pig model of myocardial ischemia and reperfusion [221]. By downregulating MMP-9 and upregulating Cx43, NRG-1 reduces the dysfunctional electrical conductivity caused by acute MI, thereby improving cardiac electrophysiological parameters and reducing the susceptibility to ventricular arrhythmia [222,223]. In conclusion, despite NRG-1’s established function in heart failure, all available evidence strongly suggests that it also has a role in acute MI in humans, making STEMI patients an additional target population for research. However, further research is required to examine the roles of NRG-1 during acute MI and its link to LVEF and other cardiac characteristics in mid- and long-term follow-up.

### 2.6. Novel Biomarkers

#### MicroRNAs

MicroRNAs (miRNAs) are small (∼22 nucleotides), single-stranded fractions of non-coding ribonucleic acids that act as micro-regulators of gene expression. They are present in a very stable state in whole blood, serum, plasma, urine and other bodily fluids. They are ideal as diagnostic or prognostic biomarkers because their levels in the blood depend on the many illnesses that they are related to [224,225,226]. The very first molecules shown to have prognostic significance in terms of mortality were miRNA-133a and miRNA-208b, which were linked to a substantial increase in all-cause death at 6 months after AMI. Other studies that looked at miRNA-208b also found this linear correlation [227,228,229]. MiRNA-192, miRNA-194 and miRNA-34 were considerably elevated in the blood of individuals who later developed heart failure, while increased levels of miRNA-155 and miRNA-380 have also been connected to cardiovascular mortality. It has been demonstrated that other microRNA molecules, including miRNA-499, are effective at predicting death at 30 days, 4 months and 1, 2 and 6 years [228,229,230,231,232]. MiRNA-145 has been shown to be able to predict cardiovascular mortality, as well as the onset of heart failure [233]. According to a recent study, greater levels of miRNA-208b and miRNA-34a in plasma can be used as indicators of left ventricular remodeling following myocardial infarction and are linked to higher mortality at 6 months, as well as a 23.1% higher probability of having heart failure. Thus, miRNA-208b seems to be a microRNA that is particular to the heart, having high levels during the acute phase of myocardial infarction and playing a prognostic role for the onset of ventricular dysfunction [234]. Increasing levels of miRNA-1, miRNA-208b and miRNA-499 in patients undergoing primary angioplasty had a detrimental effect on left ventricular ejection fraction, while miRNA-133a has also shown promise as a useful tool, being linked to large infarct areas even after reperfusion [235,236]. However, low levels of miRNA-16, miRNA-27a and miRNA-150 appear to predict ventricular remodeling, whereas elevated levels of miRNA-208b, miRNA-34a, miRNA-21 and miRNA-155 correlate negatively with the same myocardial infarction consequences [237,238,239,240]. A recent study conducted by Rincón et al. showed that the main outcome studied could be accurately predicted by miR-21-5p, miR-23a-3p, miR27b-3p, miR-122-5p, miR210-3p and miR-221-3p after a mean follow-up of 2.1 years in 311 consecutive patients hospitalized with MI [241]. Circulating microRNAs have thus displayed promising results as post-infarction prognostic biomarkers, and further research is needed to develop a risk assessment algorithm based on their levels in blood.

Studies relating prognostic biomarkers in patients with acute myocardial infarction are presented in Supplementary Materials.

Table 1 summarizes the most important studies investigating known and possible prognostic biomarkers in patients with acute myocardial infarction.

## 3. Limitations and Future Directions

Despite all the important advances in the diagnosis and treatment of patients with myocardial infarction, this still remains one of the most important causes of morbidity and mortality worldwide. In recent years, a growing number of biomarkers have been studied and proposed as potentially useful for patients with myocardial infarction, but none of them possess the characteristics of an ideal biomarker that could provide prognostic information for risk assessment of patients with AMI. However, we consider that the future holds great promise in terms of the opening of new directions of research for the identification of the best prognostic AMI biomarkers that would considerably improve the short- and long-term outcomes of these patients. Furthermore, the use of artificial intelligence algorithms for risk stratification and prognostic assessment in these patients may have an extremely important role in improving outcomes.

## 4. Conclusions

Biomarkers could become a useful tool for prognostic assessment of patients with myocardial infarction. Furthermore, biomarker research in myocardial infarction has advanced immensely over the last years, especially as a result of understanding the other processes involved in the pathophysiology of myocardial infarction, such as inflammation, myocardial stress and neurohormonal activation. Nevertheless, in the absence of randomized trials evaluating the relationship between these biomarkers and their predictive usefulness in individuals who have had an AMI, their use awaits further validation and research. Furthermore, novel biomarkers might be able to guide more individualized therapeutic strategies in order to improve prognoses.

## Figures and Tables

**Table 1:** Studies investigating known and possible prognostic biomarkers in patients with acute myocardial infarction.

Biomarker	Study	Prognostic Value
**C-reactive protein (CRP)**	Iwona et al. [18]	In patients with STEMI undergoing pPCI, CRP was associated with HF hospitalization risk and HF-related mortality in long-term follow-up (median period of 5.6 years)
	Söğüt et al. [21]	CRP/albumin ratio could predict clinical outcomes of STEMI
**Fibrinogen**	Song et al. [23]	In patients with NSTEMI undergoing pPCI, fibrinogen was a predictor of death or non-fatal reinfarction within 1 year of follow-up
	Zhao et al. [26]	In patients with STEMI undergoing pPCI, fibrinogen/albumin ratio was an independent predictor of 30-day mortality and no-reflow after pPCI
**Interleukin-6 (IL-6)**	Fanola et al. [32]	IL-6 levels, after ACS, were significantly correlated with the risk of MACEs independent of established risk predictors or other biomarkers (median follow-up of 2.5 years)
**Interleukin-37 (IL-37)**	Liu et al. [36]	In STEMI patients treated with pPCI, higher levels of IL-37 were an independent predictor for in-hospital MACEs
**B-type natriuretic peptide (BNP)**	Zubair et al. [45]	Blood-stream BNP molecular forms were related to MACE, death and HF at 6 months and 1 and 2 years follow-up in AMI patients
	Wolsk et al. [47]	In patients with ACS and diabetes mellitus, BNP was associated with significant predictions for death, CV death and HF (median follow-up of 26 months)
	Wang et al. [48]	AMI patients with high BNP levels presented low survival rates within 1 year of follow-up
	Lee et al. [49]	High initial or follow-up BNP levels were potent independent indicators for all-cause death and MACEs in AMI patients
	Hsu et al. [51]	BNP was a substantial independent predictor of LV remodeling after 6 months in AMI patients
**Mid-regional proadrenomedullin** **(MR-proADM)**	Supel et al. [54]	Elevated level of MR-proADM in plasma, measured 24 h after the diagnosis of CS, was a predictor of in-hospital mortality in patients with AMI complicated by CS
	Falkentoft et al. [56]	In patients with STEMI, increased plasma concentrations of MR-proADM were linked to elevated risks of short- and long-term all-cause mortality and cardiovascular mortality and hospital admission for heart failure, regardless of other risk factors (median follow-up of 1105 days)
**N-terminal pro-B-type natriuretic peptide (NT-proBNP)**	Gong et al. [57]	In NSTEMI patients, NT-proBNP was a powerful prognostic marker for all-cause death, hospital admission for HF and non-fatal MI or TLR (313 days median follow-up)
	Zhao et al. [59]	In STEMI patients undergoing pPCI, NT-proBNP was an independent predictor for in-hospital cardiovascular mortality, TLR, advanced HF, atrioventricular block, stroke, reinfarction and ventricular arrhythmia
	Lindholm et al. [60]	In patients with ACS, baseline values of NT-proBNP were an independent predictor for all-cause death, sudden cardiac death and death due to HF or arrhythmia
	Celebi et al. [62]	NT-proBNP assessment at admission was a good predictor for left ventricle aneurism formation in STEMI patients (6 months follow-up)
**Copeptin**	Lattuca et al. [66]	Copeptin assessed on admission in STEMI patients was an independent predictor of 1 year all-cause mortality
	Ahmed et al. [67]	Copeptin was a prognostic marker for any MACE (TLR, HF, stroke, reinfarction, cardiac death and rehospitalization for ischemic events) at 1 year of follow-up in NSTEMI patients
**Platelet-related biomarkers**	Avci et al. [74]	In STEMI patients, increased MPV values during hospitalization were correlated with long-term mortality
	Chang et al. [75]	High MPV levels were associated with increased risk of MACEs (all-cause mortality, time to recurrent ACS, stroke and TLR) in ACS patients (median follow-up of 2.4 years)
	Çanga et al. [77]	MPV was an independent predictor of MACEs in short-term follow-up (cardiovascular death and non-fatal reinfarction within 30 days) in young STEMI patients
	Kurtul et al. [79]	MPV was a predictor for short-term mortality and no-reflow phenomena in STEMI patients
	Chunyang et al. [81]	MPV/PC ratio was a long-term adverse outcome predictor in STEMI patients (30 months of follow-up)
	Ösken et al. [82]	In STEMI patients, MPV/PC ratio was correlated with long-term ST and mortality (5 years of follow-up)
**Troponins**	Zeljković et al. [85]	cTnT was a predictor for LV systolic dysfunction (<50%) within 1 year of follow-up in STEMI patients
	Mohammad et al. [86]	In STEMI patients, the hs-cTnT level predicted long-term LV dysfunction (12 months of follow-up)
	Ndrepepa et al. [90]	In patients with STEMI undergoing pPCI, admission or peak post-procedural hs-cTnT were independently linked with the probability of 3 year death
	Harada et al. [91]	Post-procedural hs-TnT was independently related with higher risk of death up to 1 year after PCI in individuals with NSTEMI who received early PCI
**Creatine kinase-MB (CK-MB)**	Johannes et al. [95]	CK-MB was a risk factor for HF onset after STEMI (median follow-up of 6.7 years)
	Ndrepepa et al. [98]	Peak post-procedural CK-MB was a predictor of 3 year mortality
	Hsu et al. [51]	CK-MB was an independent predictor of LV remodeling after 6 months in AMI patients
**Cystatin C (cysC)**	Cheng et al. [109]	CysC was a predictor for no-reflow phenomena in STEMI patients undergoing pPCI
	Lou et al. [110]	CysC was a predictor for MACE (cardiovascular mortality and all-cause mortality) in AMI patients
	Brankovic et al. [111]	Independently of the GRACE risk score, cysC levels predicted death or recurrence of ACS during the first year
	Barbarash et al. [112]	CysC was a predictor of adverse cardiovascular outcomes within 3 years of follow-up in STEMI patients
	Correa et al. [115]	CysC was a predictor of adverse cardiovascular outcomes in ACS patients (median follow-up of 2.5 years)
	Mao et al. [116]	CysC was an independent predictor of MACEs (cardiac death, non-fatal MI, TLR, HF, non-fatal stroke) in NSTEMI patients within 12 months of follow-up
	Chen et al. [117]	High cysC levels at admission were an independent predictor of cardiac mortality and long-term all-cause mortality in STEMI patients (median follow-up of 40.7 months)
**Endothelial cell-related biomarkers**	Ziaee et al. [131]	Endocan was an independent predictor for MACEs (in-hospital death, HF and recurrent ischemia) comparable with that of the TIMI risk score in ACS patients
	Dogdus et al. [134]	Endocan was an independent predictor for no-reflow phenomena in STEMI patients
**Aspartate transaminase (AST)**	Steiniger et al. [138]	De-Ritis ratio was a strong independent predictor for long-term mortality in AMI patients (median follow-up of 8.7 years)
**Galectin-3 (Gal-3)**	Giuseppe Di Tano et al. [154]	In patients with a first anterior STEMI treated with pPCI, Gal-3 levels were a strong independent predictor of long-term all-cause death and HF hospitalization (median follow-up of 22 months)
	Rabea et al. [155]	Gal-3 was an independent predictor of HF and mortality after an AMI (median follow-up of 5.4 years)
	Stanojevic et al. [162]	STEMI patients with high Gal-3 levels presented 4.4 times greater risk of developing AF
	Agata et al. [164]	Gal-3 was an independent predictor for HF onset at 1 year of follow-up in STEMI patients treated with pPCI
	Gagno et al. [156]	Gal-3 was an independent predictor for 1 year all-cause mortality but not for AMI or angina pectoris
**Soluble suppression of tumorigenicity 2 (sST2)**	Somuncu et al. [167]	Within 1 year of follow-up in patients with MI, high levels of sST2 were a strong predictor of poor CV outcomes, including CV death and heart failure
	Hartopo et al. [168]	sST2 levels were an independent predictor of adverse cardiac events (cardiac death, acute HF, reinfarction, resuscitated ventricular arrythmias, cardiogenic shock) during acute intensive care for STEMI
	Jenkins et al. [171]	Higher values of sST2 after an AMI were correlated with increased risk of HF and death over a long-term follow-up period (median period of 5 years)
	Shiru et al. [176]	sST2 was a predictor marker for impaired myocardial reperfusion in STEMI patients treated with pPCI
	Yu et al. [174]	Elevated sST2 levels at admission were independent predictors for 1 year MACEs in STEMI patients
	Liu et al. [175]	In patients with STEMI undergoing PCI, sST2 was found to be an independent predictor for MACEs (all cause death, a non-fatal MI and HF) and mortality (12 months of follow-up)
**Growth differentiation factor-15 (GDF-15)**	Peiró et al. [178]	Concentrations greater than 1800 ng/L were linked to an elevated risk of all-cause mortality, MACE, hospitalization for HF and cardiovascular death
	Li et al. [179]	
	Zelniker et al. [180]	
**Syndecan-1 (Sdc1)**	Wernly et al. [184]	Sdc1 > 120 ng/mL was independently linked with death at 6 months
**Circulating LIPCAR**	Yan et al. [186]	LIPCAR may be a biomarker of early HF following AMI
	Li et al. [187]	In STEMI, greater levels of LIPCAR were found to be independent predictors of significant adverse cardiovascular events
**Thrombospondin-1 (TSP-1)**	Liao et al. [191]	TSP-1 was an independent risk factor for atrial arrhythmias in patients with AMI
**Uric acid (UA)**	Lazaros et al. [197]	In ACS, peak admission UA levels could predict both 30 day and 1 year mortality Hyperuricemia has been linked to an increased risk of 2 and 5 year all-cause mortality in STEMI patients following PCI, with the best cut-off value to predict MACE in young patients with NSTEMI being 5.2 mg/dL
	Tang et al. [198]	
	Kaya et al. [199]	
	Çanga et al. [200]	
**Neuropeptide-Y (NPY)**	Herring et al. [208]	NPY was independently associated with coronary microvascular dysfunction, increased cardiac injury and decreased LV ejection fraction 6 months after an acute event and with subsequent heart failure and mortality over an average follow-up of 6.4 years
	Gibbs et al. [211]	
**MicroRNAs (miRNAs)**	Widera et al. [227]	miRNA-133a and miRNA-208b were linked to an important rise in all-cause death at 6 months after an AMI
	Goretti et al. [228]	miRNA-499 was found to be effective at predicting death at 30 days, 4 months and 1, 2 and 6 years
	Xiao et al. [229]	
	Olivieri et al. [230]	
	Matsumoto et al. [231,232]	
	Dong et al. [233]	miRNA-145 has been shown to be able to predict cardiovascular mortality, as well as the onset of heart failure
	Wang et al. [234]	miRNA-208b and miRNA-34a can be used as indicators of LV remodeling following myocardial infarction and are linked to higher mortality at 6 months, as well as a 23.1% higher probability of having HF
	Rincón et al. [241]	miR-21-5p, miR-23a-3p, miR27b-3p, miR-122-5p, miR210-3p and miR-221-3p could accurately predict hospital admission for HF or cardiovascular death after a mean follow-up of 2.1 years

ACS, acute coronary syndrome; AMI, acute myocardial infarction; CRP, C-reactive protein; cysC, cystatin C; Gal-3, galectin-3; HF, heart failure; hs-cTnT, highly sensitive cardiac troponin; LV, left ventricular; MACE, major cardiovascular event; microRNA, microribonucleic acid; MPV, mean platelet volume; MR-proADM, mid-regional proadrenomedullin; LIPCAR, a long noncoding ribonucleic acid; NT-proBNP, N-terminal pro-B-type natriuretic peptide; NSTEMI, non-ST-elevation myocardial infarction; PC, platelet count; pPCI, primary percutaneous coronary intervention; STEMI, ST-elevation myocardial infarction; TLR, target lesion revascularization.

## Data Availability

The data presented in this study are available on request from the corresponding author.

## Supplementary Materials

The following supporting information can be downloaded at: https://www.mdpi.com/article/10.3390/ijms23169168/s1.
