# Supplementary material for: From Classic to Modern Prognostic Biomarkers in Patients with Acute Myocardial Infarction
Source: Int J Mol Sci. 2022 Aug 15;23(16):9168. doi: 10.3390/ijms23169168 (PMC9409468; doi:10.3390/ijms23169168)
Supplement: Supplementary file 1 [file ijms-23-09168-s001.zip › ijms-1831042-supplementary.pdf]

Studies relating prognostic biomarkers in patients with acute myocardial infarction

Scheme	Study type	Characteristics	Results	Prognostic Value
**BIOMARKERS OF INFLAMMATION**				
**C-REACTIVE PROTEIN (CRP)**				
Iwona et al.	Prospective observational cohort study	204 patients with first STEMI;<br>Median period of 5.6 years (4.9-6.3);<br>24 patients - hospitalization for HF vs 180 patients - without hospitalization for HF;	• CRP at admission – no prognostic value regardless the presence of HF (2.37 mg/L vs 1.68 mg/L, $p = 0.075$);<br>• CRP values during hospitalization for STEMI and one month after discharge ($CRP_{1M} \geq 2$ mg/L) were higher in patients requiring HFH compared to those with no HFH (19.94 mg/L vs 9.21 mg/L, $p < 0.001$; 2.57 mg/L vs 1.54 mg/L, $p = 0.01$);	• Risk for HF hospitalization and HF-related mortality long-term follow-up;<br>• $CRP_{1M} \geq 2$ mg/L exhibited higher risk of HFH long-term;
Fu et al.	Large Health Care-Based Study	12905 patients with AMI (62% men);<br>Mean age 73 years and 3 years post AMI;<br>Median period of 3.2 years (1.8-4.7);<br>35% had eGFR < 60 ml/min/1.73 m^2;	• Patients with hs-CRP ≥ 2 mg/L were at higher risk of CKD progression and AKI compared to the lower group;	• In post-AMI patients, increased hs-CRP values were associated with a consecutive risk of AKI and CKD progression regardless of kidney function;
SÖĞÜT et al.	Prospective study	116 patients with STEMI (86.2% men);<br>Mean age 56.47 ± 11.42 years;	• Mean CRP/albumin ratio, cTnI and mean number of coronary vessels affected were greater in non-survivors than in survivors ($p = 0.006$; $p = 0.004$; $p = 0.007$);<br>• ≥ 2 vessels affected – the most important predictor of mortality ($p = 0.009$);	• CRP/albumin ratio could predict clinical outcomes of STEMI;<br>• ≥ 2 vascular lessions contributed to a 2-fold increase in mortality rate in STEMI patients;
**FIBRINOGEN (FIB)**				
Song et al.	Prospective cohort study	1211 patients with NSTE-ACS<br>Two groups - low fibrinogen and high fibrinogen group	• Elevated baseline fibrinogen level (> 3.49 mg/dl) is an independent predictor for death/nonfatal reinfarction ($p = 0.035$);<br>• Prognostic performance of fibrinogen - equivalent to GRACE system in predicting death or nonfatal reinfarction;	• Prediction of death or non-fatal reinfarction within one year in those with NSTE-ACS undergoing PCI;

Zhao et al.	Retrospective analytic-cross sectional study	510 STEMI patients with successfully primary PCI between September 2016 – May 2018; No-reflow phenomenon – was post-PCI TIMI flow grade of 0,1 and 2;	• Compared to normal reflow group, no-reflow one presented higher levels of fibrinogen/albumin ratio (FAR) (9.72 vs 13.31, p < 0.001); • ROC analysis demonstrated that FAR had 69.42% specificity and 79.59% sensitivity for the formation of no-reflow status (95% CI 0.786-0.852, p < 0.001); • Univariate Cox regression analysis model revealed that FAR is a predictive tool of 30-day mortality after pPCI (HR 1.409, 95% CI 1.347-1.473, p < 0.001);	• FAR is an independent predictor of 30-day mortality and no-reflow after pPCI;
		**GALECTIN-3 (Gal-3)**		
Giuseppe Di Tano et al.	Prospective, observational, single-center study	103 consecutive patients with a first anterior STEMI treated by pPCI (72% men); Mean age 65 years (56-76); Median follow-up of 22 months (3-30);	• Gal-3 independently predicted the combined end-point (HR 1.11; 95% CI: 1.05-1.17; per 1 ng/ml increase); • Gal-3 levels above or below 16.8 ng/ml resulted in 42.3 and 93.5% of event-free survival rates (p < 0.001);	• Gal-3 levels after a PCI are a strong independent predictor of long-term all cause-death and HFH;
Rabea et al.	Prospective population-based incidence MI cohort study	1342 patients with AMI (61.3 male); 71.8% NSTE-ACS; Mean age 67.1 years; Median follow-up 5.4 years (3.5);	• After adjusting for key clinical characteristics, there was a mortality increasement by 30% and 15% increased risk for HF, for every 10-unit rise in Gal-3 (HR 1.30, 95% CI 1.24-1.36; HR 1.15, 95% CI 1.09-1.22);	• Gal-3 is an independent predictor of HF and mortality after an AMI;
Turan et al.	Cross-sectional study	98 consecutive STEMI patients undergoing pPCI separated in ST regression (STR) group and one in incomplete ST regression;	• A multivariate logistic regression demonstrated that in STEMI patients, Gal-3 concentration presented an important predictive role of incomplete STR (OR = 0.212; 95% CI = 0.084-2.752; p = 0.017); • The multivariate linear regression analysis showed an association between Gal-3 and SYNTAX score (β = 0.212, p = 0.037);	• Gal-3 is an independent predictor for incomplete STR hence, it may be a useful aid in determining patients with an increased risk for insufficient myocardial perfusions

				and poor clinical outcome;
Stanojevic et al.	Observational study	58 AMI patients with AF and 38 AMI patients without AF used as a control group;<br>14 months follow-up;	• Patients with AF presented higher levels of Gal-3 ($p < 0.05$) than those without AF ($p < 0.01$);<br>• High Gal-3 levels are associated with 4.4 times greater risk of developing AF;<br>• Gal-3 > 7.57 ng/ml – independent predictor for AF in AMI;	• Independent predictor for AF;
Przemyslaw et.al	Pilot study	110 AMI patients treated by pPCI hospitalized between 2012-2014 and 100 healthy volunteers;	• In a multivariate analysis Gal-3 levels greater than 9.2 ng/ml at discharge was linked to a nine-fold increased risk of composite endpoint occurrence (p = 0.0005, OR = 9.47, 95% CI 2.60-34.45);<br>• 1 ng/ml increase in Gal-3's concentration determined an increasement of the composite endpoint occurrence (subsequent MI, Re-PCI, CABG, stroke) by 14% (p = 0.004, OR=1.145, 95% CI 1.04-1.26);	• Elevated levels of Gal-3 were corelated with composite endpoint appearance during long-term follow-up;
Agata et al.	Prospective, observational study	111 STEMI patients treated with pPCI between October 2014 – April 2017;	• In a multivariate analysis Gal-3 was a predictor of the primary endpoint (HF onset at one-year follow-up (p = 0.02, HR 1.61, 95% CI 1.07-2.42);	• Gal-3 is an independent predictor for HF onset at one-year follow-up;
Gagno et al.	Prospective study	469 AMI patients;<br>12 months follow-up;	• A multivariable Cox proportional hazards regression analysis showed that Gal-3's levels are statistically associated with a higher risk for one-year all-cause death (p = 0.002 HR = 3.5; 95% CI (1.54-7.5);	• Gal-3 is an independent predictor for one-year all-cause mortality but not for AMI or angina pectoris;
		**INTERLEUKIN 6 (IL-6)**		
Fanola et al.	Randomly selected biomarker cohort from a randomized, double-blind, placebo controlled, multicenter event-driven trial	4939 patients with ACS;<br>Median follow-up 2.5 years;<br>Baseline characteristics were determined by IL-6 quartiles;	• Subjects with the highest IL-6 levels presented more than two-fold greater risk of cardiovascular death or HF (adj HR 2.29, 95% CI 1.6-3.29), a 57% higher risk of MACE (adj HR Q4:Q1 1.57, 95% CI 1.22-2.03) and higher risk of AMI (adj HR 1.39 95% CI 1.01-1.93);	• IL-6 levels, after ACS, are significantly corelated with the risk of MACE independent of established risk predictor or another biomarker;
		**INTERLEUKIN 37 (IL-37)**		

Liu et al.	Prognostic study	125 STEMI patients treated with pPCI admitted from July 2014 to December 2016 divided in two groups;	• Patients with greater levels of IL-37 (≥ 341.1 pg/ml) had a longer time hospitalization, a lower LV function and a greater risk of non-fatal MI, TLR, acute HF and in-hospital mortality (OR 3.652 95% CI 1.113-11.983);	• Higher levels of IL-37 were an independent predictor for in-hospital MACE ($p < 0.05$);
**PROCALCITONIN (PCT)**				
J Dai et al.	Case-control study	1200 patients divided in three groups (400 with STEMI, 400 with unstable angina pectoris (UAP) and 400 control) enrolled between 1 January 2014 and 31 December 2015 with monthly follow-up for 12 months;	• In STEMI patients, greater values of admission PCT (≥ 1.4 μg/L), peak PCT (≥ 5.1 μg/L) and average PCT (≥ 3.9 μg/L) were corelated with about 50%, 150% and 150% increased risk for MACE (HR:1.46, 95%CI: 1.18–1.99; HR: 2.57, 95%CI: 1.99–3.52; HR: 2.36, 95%CI: 1.81–3.00, $p < 0.001$);	• PCT levels might be an independent predictor for HF, UAP/AMI recurrence, cardiac death and severe arrhythmia;
**SOLUBLE SUPPRESION OF TUMORIGENICITY 2 (sST2)**				
Somuncu et al.	Prospective study	380 AMI patients grouped in low sST2 (n = 264, mean age: 60.0 ± 12.1 years) and high sST2 groups (n = 116 mean age: 60.5 ± 11.6 years); 12 months follow-up;	• At one-year follow-up the HF rate (8.6% vs 3.4%, p = 0.032), mortality (15.5% vs 4.9%, p = 0.001) and cardiovascular adverse outcomes ($p < 0.001$) were higher in the high sST2 group; • In a multivariate Cox regression analysis, high sST2 levels are predictor for 1-year cardiovascular mortality (HR 2.263; 95% CI 1.124–4.557; p = 0.022);	• Within 1-year follow-up in patients with MI, high levels of sST2 are a strong predictor of poor CV outcomes, including CV death and heart failure;
Hartopo et al	Cohort study	105 patients (95 STEMI and 10 control) divided in two groups (supramedian and inframedian) enrolled between April 2014 and January 2015 followed until discharge;	• After a multivariate analysis the supramedian group sST2 was independently corelated with higher incidence of adverse cardiac events (Adj OR 6.27; 95% CI 1.33-29.47, p = 0.020)	• The sST2 levels are independently predictor of adverse cardiac events (cardiac death, acute HF, reinfarction, resuscitated ventricular arrythmias, cardiogenic shock) during acute intensive care of STEMI;

Melvin et al.	Prospective cohort study	122 ACS patients with or without diabetes; Median follow-up 180 days;	• In ACS patients, AUC for anticipating MACE occurrence with sST2 was 0.72 (p = 0.04; 95% CI (0.58-0.91)) and for predicting mortality was 0.85 (p = 0.02; 95% CI (0.74-0.96));<br>• The risk of MACE (p = 0.004) and mortality (p = 0.006) were higher in the ACS patients with elevated levels of sST2;<br>• In the fully adjusted Cox regression model, ACS patients with a sST2 level greater than 36.5 ng/ml had a 5.8-fold (p = 0.032) increased risk of MACE;	• In the regression analysis, elevated levels of sST2 remained an independent predictor of MACE;
Jenkins et al.	Prospective cohort study	1401 AMI patients; 5 years follow-up; Three tertiles: tertile 1: 0 < sST2 ≤ 37 ng/mL, tertile 2: 37 < sST2 ≤ 72.3 ng/mL, and tertile 3: sST2 > 72.3 ng/mL;	• After adjustment for other prognostic factors, sST2 levels in tertiles 2 and 3 were associated with an independent 1.7-fold (HR 1.74, 95 % CI 1.22-2.50) and almost 3-fold (HR 2.94, 95 % CI 2.08-4.16) higher risk of mortality, respectively (p < 0.001);<br>• After adjustment for age, sex, Killip class, maximum Troponin T, HF prior to AMI, Charlson comorbidity index, sST2 levels in tertiles 2 and 3 still increased the risk of heart failure by about 1.7-fold (HR 1.67, 95 % CI 1.17-2.37) and 3-fold (HR 2.98, 95 % CI 2.11-4.22), (p < 0.001);	• Higher values of sST2 after an AMI are corelated with a increased risk of HF and death over a long-term period follow-up;
Mzoughi et al.	Prospective longitudinal study	74 AMI patients enrolled between April and October 2016; 54% - STEMI; 46% - NSTE-ACS;	• In a multivariate analysis sST2 was associate with MACE (RR = 2, p = 0.04);<br>• The 35 ng/ml ST2 cutoff value showed a sensitivity of 95%, a specificity of 30% (AUC = 0.672, CI 0.546-0.798, p = 0.024), a negative predictive value of 100%, and a positive predictive value of 33%;	• ST2 is a novel predictive biomarker for in-hospital morbidity and death in AMI patients;
Shiru et al.	Prospective study	121 STEMI patients enrolled between January 2016 and August 2017 and followed during their hospitalization;	• In a multivariable logistic regression analysis sST2 was found to be an independent predictor of impaired myocardial reperfusion (OR 12.318, 95% CI 4.567-33.220, p < 0.001);<br>• The area under the curve for sST2 was 0.849, with an optimal cut-off value of 2.003 ng/mL, with sensitivity of 89.2% and a specificity of 67.9%, according to ROC curve analysis;	• sST2 is a predictor marker for impaired myocardial reperfusion in STEMI patients treated by pPCI;
Yu et al.	Prospective observational study	425 STEMI patients treated by pPCI from January 2011 to January 2015; One year follow-up after PCI;	• After adjusting for other risk factors, Cox regression analysis showed that increased serum sST2 (> 75.8 ng/mL mean value, adj HR 2.098, 95% CI 1.008-4.367, p = 0.048) at admission are independently predictors for MACCE (cardiovascular death, non-fatal MI, non-fatal stroke, and ischemia-driven revascularization) within first year of follow-up;	• Elevated sST2 levels at admission are independent predictors for one-year MACCE;

		Mean age 59.1 ± 13.1 (men 84%);		
Liu et al.	Prospective observational study	311 STEMI patients; 12 months follow-up;	• A higher baseline sST2 value greater than 58.7 ng/mL was independently corelated with mortality (HR 5.01, 95% CI 1.02-16.30, p = 0.048), associating a sensitivity of 70% and a specificity of 77.2%;<br>• After adjusting for prognostic markers, an increased sST2 level was associated with a two-fold higher risk of MACEs defined as all cause death, a non-fatal MI and HF (HR 2.23, 95% CI 1.20-6.78, p = 0.001);	• In patients with STEMI undergoing PCI, sST2 showed to be an independent predictor for MACEs and mortality;
**Scheme**	**Study type**	**Characteristics**	**Results**	**Prognostic Value**
			**BIOMARKERS OF NEUROHORMONAL ACTIVATION**	
			**B-TYPE NATRIURETIC PEPTIDE (BNP)**	
Zubair I. et al.	Prospective study	1078 AMI patients; Mean age 67 years (57-77); Follow-up for two years; Three isoforms: BNP 5-32; BNP 4-32; BNP 3-32;	• In a Kaplan-Meier survival analysis, with patients divided into three groups, after using the GRACE score for risk prediction, BNP 3-32 was associated with a 2.6-fold-increase risk of death/MI at six months for the middle and higher risk group as compared to the low risk one (p < 0.001);<br>• BNP molecular forms were univariate predictors of death/HF, MACE and death/MI at 6 months, one year and two years (all p < 0.001);	• Blood stream BNP molecular forms are related with MACE, death and HF at 6 months, one- and two-years follow-up;<br>• BNP 5-32 can be used a secondary risk stratification marker in selecting high risk patients for outcome at 6 months follow-up after GRACE score risk classification;
Carvalho et al.	Prospective study	167 STEMI patients with Killip I class; Follow-up for one year;	• At 30 days, the probability of developing symptomatic HF or presenting LVEF ≤ 40% was raised by 8.7 (95% CI 1.10-662, p = 0.046) for every 100 pg/dl increase in BNP-change;<br>• BNP-change was linked with sudden death/MI at 30 days in both univariate and variate Cox analysis (OR 1.032 for each increment of 10 pg/dl, 95% CI 1.03-1.052, p < 0.001), but not BNP-D1;<br>• The addition of BNP-change to the GRACE score enhanced risk classification (C-statistic=0.831, p=0.001);	• Only BNP-change after AMI was corelated with impaired short- and long-term outcomes and enhanced the risk reclassification;

Wolks et al.	Prospective study	5525 ACS patients with diabetes mellitus type 2; 26 months follow-up;	• In univariate analysis, a BNP level of 228 pg/ml divided patients into a lower vs higher group at risk of developing HF (sensitivity: 0.62, specificity: 0.77, Youden index: 0.39);<br>• A BNP value of 500 pg/ml in adjusted Cox model regression identified individuals at reduced vs higher risk of future HF (HR 3.0 [2.1–4.1], P<0.0001)<br>• When BNP was included to risk models, it improved C statistics for each outcome studied, with the biggest increases in mortality (0.77–0.82, p < 0.001), cardiovascular death (0.77–0.83, p < 0.001), and HF (0.84–0.87, p < 0.001).	• In patients with ACS and diabetes mellitus, BNP was associated with a significant prediction for death, CV death and HF;
Wang et al.	Prospective observational study	180 AMI patients treated by pPCI; Mean age 61.41 ± 8.9 years; Follow-up for one year;	• The serum concentrations of BNP were considerably different between MACE and MACEs free group (433.27 ± 95.64 pg/ml vs 253.99 ± 66.68 pg/ml, p < 0.05) being an independent risk predictor of MACEs (p < 0.05) with an area under curve of 0.902 (95% CI 0.86-0.95, p < 0.05);<br>• According to Pearson correlation analysis, BNP levels displayed favorable relationships with Gensini score (r = 0.6, p < 0.05);<br>• At one-year follow-up, overall survival rate was lower in AMI patients with high serum levels of BNP compared to those with lower ones (p < 0.05);	• Patients with high BNP levels within one-year follow-up, have low survival rates;
Lee et al.	Prospective single-center study	442 AMI patients (71% male); Median follow-up 441 days (IQR: 362-861]; Mean age 65.0 ± 12 years;	• After adjusting for risk prediction factors, follow-up BNP levels were an independent predictor for MACE (OR, 1.43; 95% CI, 1.101–1.858) and for all-cause mortality (OR, 2.265; 95% CI, 1.455–3.527), but not the initial BNP level;<br>• However, high initial BNP levels were corelated with all-cause mortality (OR, 3.465; 95% CI: 1.122–10.700) and MACE (OR: 2.084, 95% CI: 0.945–4.598);	• High initial or follow-up BNP levels were a potent independent indicator for all-cause death and MACE in AMI patients;
Shindo et al.	Sub-study of a prospective randomized multicenter trial	870 AMI patients;	• High BNP values are associated with an increased risk of all-cause hospitalization due to a non-fatal MI, worsening of HF or due to PCI and CABG for stable angina (all p < 0.0001);	• In patients with AMI and impaired glucose tolerance, high serum BNP concentrations are predictor of worse cardiovascular outcomes;

Hsu et al.	Prospective study	110 AMI patients;<br>6 months follow-up;	• RBNP13 remained a significant independent predictor of 6-month LV remodeling (odds ratio = 0.967, P = 0.003) after controlling for clinical, laboratory, and angiographic factors, with a cut-off value of $RBNP_{13}$ of 53.2% (AUC = 0.764, P < 0.001);<br>• It has a negative predictive value of 87.5% and a positive predictive value of 53.6%;	• $RBNP_{13}$ is a substantial independent predictor of LV remodeling after 6 months;
**MID-REGIONAL PROADRENOMEDULLIN (MR-proADM)**				
Supel et al.	Prospective observational study	47 ACS patients complicated by cardiogenic shock (CS) and underwent PCI (60% men)	• MR-proADM 2, in univariate logistic regression, the ROC curve analysis revealed that was a predictor of in-hospital mortality in patients with AMI complicated by CS (AUC 0.774, p1 40.0017, Youden index 0.58), but not in multivariable analysis;	• Elevated plasma level of MR-proADM, measured 24 hours after the diagnosis of CS, is a predictor of in-hospital mortality in patients with AMI complicated by CS;
Wegiel et al.	Prospective study	80 STEMI patients treated by pPCI;<br>6 months follow-up;	• In a univariate regression and multivariable first model, MR-proADM was an independent predictor of revers remodeling (OR 1.6 per 1 ng/ml, 95% CI 1.02-2.7, p < 0.04 and OR 3.4 per 1 ng/ml, 95% CI 1.58-9.2, p = 0.001);<br>• After ROC analysis, the cut-off value for MR-proADM was 3.95 ng/ml, AUC = 0.68 in predicting LV revers remodeling (> 10% reduction in LV end-systolic volume);	• MR-proADM is a biomarker of reverse remodeling in AMI patients;
Falkentoft et al.	Post-hoc sub-study of DANAMI-3 trial (three randomized controlled multicenter trials)	1122 STEMI patients;<br>Median follow-up 1105 days (917-1281);	• In a univariate analysis, elevated levels of MR-proADM were associated with increased risk of 30-day mortality or cardiovascular mortality (HR 3.93 per doubling; 95% CI, 2.51–6.14; p < 0.0001), the link remaining significant after adjusting for age and sex (HR, 3.14; 95% CI, 1.86–5.33; p < 0.0001) and multivariable analysis (HR 2.67; 95% CI, 1.01–7.11; p = 0.049);<br>• A doubling of MR-proADM concentrations was linked with a 3.43 increase in HR (95% CI, 2.67–4.41; p < 0.0001) in long-term all-cause death, according to univariate analysis, with the same relationship after adjusting for age and sex (HR, 2.59; 95% CI, 1.91–3.52; p < 0.0001) and multivariable adjustments (HR, 3.23; 95% CI, 1.97–5.29; p < 0.0001);	• In patients with STEMI, increased plasma concentrations of MR-proADM were linked to an elevated risk of short- and long-term all-cause mortality and cardiovascular mortality, hospital admission for heart failure, regardless of other risk factors.

			• For 30-day and 3-year mortality, the area under the curve for MR-proADM was 0.77 and 0.78, respectively;	
**N-TERMINAL PRO-B-TYPE NATRIURETIC PEPTIDE (NT-proBNP)**				
Gong et al.	Observational cohort study	1357 NSTE-ACS patients;<br>12 months follow-up (average 313 days);	• The Cochran-Armitage test demonstrated that high levels of NT-proBNP were associated with a greater rate of MACE, all-cause death and hospital admission for HF (49.67%, 21.17% and 18.54%, all p < 0.0001);<br>• NT-proBNP was shown to be an independent risk factor for composite MACEs [medium vs. low, HR: 2.19 (1.45–3.32), P=0.0002]; [high vs. low, HR: 3.07 (1.78–5.29), P0.0001], as well as all-cause mortality and heart failure in the multivariable Cox model, with a higher prognostic value in patients over 60 years old or LVEF < 40%;	• NT-proBNP is a powerfull prognostic marker for all-cause death, hospital admission for HF or UAP, non-fatal MI or TLR;
Zhao et al.	Retrospective study	216 STEMI patients undergoing pPCI;	• In a multivariate logistic regression analysis, NT-proBNP (OR 1.052, 95% CI 1.029-1.076, p = 0.023) along with fQRS ≥ 3 leads or on anterior leads, were independently associated with in-hospital MACEs;<br>• In the fQRS (+) group, the rate of advanced heart failure in hospital was substantially greater, with a sensitivity 80.8% and specificity 70% in fQRS, for NT-proBNP levels ≥ 801.5 pg/ml;<br>• The AUC for predicting fQRS using the NT- proBNP level was 0.809, fQRS being a prognostic marker of reduced regional ventricular systolic function;	• In STEMI patients undergoing pPCI, NT-proBNP is an independent predictor for in-hospital cardiovascular mortality, TLR, advanced HF, atrioventricular block, stroke, reinfarction, ventricular arrhythmia;
Lindholm et al.	Randomized clinical trial	17095 patients with ACS enrolled between October 2006 and July 2008;<br>Median age 62.0 years (54.0-71.0)	• Increased levels of NT-proBNP were associated with a four-fold elevated risk of sudden cardiac death or arrhythmia (HR = 3.89, 95% CI 2.29-6.60), with an eight-fold increasing risk for death secondary to HF (HR = 8.20, 95% CI 2.60-25.88);<br>• Both in the unadjusted analyses and in the adjusted ones, NT-proBNP remained the most powerfull predictor for all-cause mortality (HR 3.47; 95% CI 2.75-4.38 vs HR 2.96; 95% CI 2.33-3.76);	• In patients with ACS, baseline values of NT-proBNP were independent predictor for all-cause death, sudden cardiac death or death due to HF or arrhythmia;
Zhang et al.	Retrospective study	1823 STEMI patients;<br>103 patients presented LV aneurysm (LVA)	• High peak NT-proBNP levels were independent predictor of early onset LVA formation in patients with STEMI (OR 1.08, 95% CI 1.01-1.16, p = 0.031);	• NT-proBNP is a predictor factor for early LVA formation in patients with STEMI

		compared with 206 patients without LVA;		associating regional wall motion abnormality;
Celebi et al.	Prospective study	1519 STEMI patients; 6 months follow-up;	• NT-proBNP admission levels greater than 400 pg/ml were independent predictor for LVA development after AMI, its concentration being statistically higher in patients who develop LVA (523.5 ± 231.1 pg/mL vs. 192.3 ± 176.6 pg/mL, respectively, $p < 0.001$);	• NT-proBNP assessment at admission is a good predictor for LVA formation;
		**COPEPTIN**		
Roczek-Janowska et al.	Prospective study	100 AMI patients (61% men); Mean age 63 ± 7 years; Follow-up 12 months;	• The presence of elevated copeptin on the fourth or fifth day of hospitalization was found to be a predictor of significant adverse cardiovascular events ($p = 0.0445$); • In a receiver operating characteristic (ROC) analysis, a rise in copeptin level from admission to day 4/5 was linked to the need for unscheduled coronary revascularization (AUC = 0.639; 95% CI 0.504–0.773; $p = 0.0430$); • In a multivariate analysis, copeptin levels on the 4th/5th day of hospitalization and LVEF determined by transthoracic echocardiography were the sole predictors of severe adverse cardiac events during follow-up ($p = 0.024$ and $p = 0.001$);	• The measurement of copeptin concentration on admission and on the 4th/5th day of hospitalization in patients with AMI treated with PCI might have predictive significance for MACE;
Lattuca et al.	Prospective study	401 STEMI patients treated by pPCI; Follow-up 365 days;	• Patients who died during the first 30 days of follow-up had 10 times the amount of copeptin as survivors (median 211.1 pmol/L; IQR [102.1–400.1] vs 30.7 pmol/L; IQR [10.7–93.1]; $p < 0.01$) but also after one-year follow-up (median 154.8 pmol/L; IQR [63.9–304.8] vs 30.3 pmol/L; IQR [10.8–93.5]; $p < 0.01$); • The fourth quartile of copeptin levels was significantly linked with a greater probability of death at one year (HR=4.6; 95% CI 2.8–7.6, $p < 0.001$), and after adjusting for possible confounders, it remained an independent predictor (adjusted HR 3.1; 95% CI 1.5–6.2; $p = 0.001$);	• Copeptin assessed on admission in STEMI patients is an independent predictor of one-year all-cause mortality;
Ahmed et al.	Prospective observational study	79 patients (40 UAP, 39 NSTE-ACS); One-year follow-up;	• Patients who experienced MACEs or coronary revascularization throughout the follow-up period had substantially higher baseline admission copeptin levels (p-value 0.001 for each); • The univariable logistic regression analysis identified plasma copeptin as a predictor for overall MACEs (HR: 0.1, 95% CI =0.01–0.5, $p = 0.007$) and in a multivariate analysis copeptin was found to be a separate	• Copeptin is a prognostic marker for any MACEs (TLR, HF, stroke, reinfarction, cardiac death and rehospitalization for

			• predictor of MACEs (HR: 0.01, 95% CI: 0.0–0.8, p = 0.04), along with a GRACE risk score;	ischemic events) at one-year follow-up;
Pamukcu et al.	Prospective observational single center study	122 STEMI patients (73.8% male); 6 months follow-up; Mean age 57.6 ± 10.7 years;	• Copeptin levels were negatively associated with early and 6-month LV EF (r = –0.299 at early stage and r = –0.410 at 6-month) and global longitudinal strain (r = –0.459 at early stage and r = –0.662 at 6-month);	• After a STEMI diagnosis, higher plasma copeptin levels were linked to lower LV systolic function, determined by strain echocardiography;
**Scheme**	**Study type**	**Characteristics**	**Results**	**Prognostic Value**
		**BIOMARKERS OF MYOCARDIAL NECROSIS**		
		**PLATELET RELATED BIOMARKERS**		
Avci et al.	Retrospective study	480 STEMI patients treated with pPCI; Median follow-up 65.9 months (41.9-80.4);	• A high ΔMPV was an independent predictor of all-cause mortality (HR: 1.301 95% CI 1.070–1.582, p = 0.008) with AUC in a multivariable mode of 0.781 (95% CI:0.731–0.832, p < 0.001);	• In STEMI patients, increased MPV values during hospitalization was corelated with long-term mortality;
Chang et al.	Prospective hospital-based study	1094 ACS patients + 472 non-ACS patients; Mean follow-up 2.4 years; Divided in three groups: low MPV, medium MPV and high MPV;	• MACEs were substantially more common in the high MPV group (> 9.0 fl, n = 305) than in the low MPV group (p = 0.017);	• High MPV levels are associated with increased risk of MACE (all-cause mortality, time to recurrent ACS, stroke and TLR);
Çanga et al.	Retrospective study	349 young STEMI patients (90% male); Mean age 36.4 ± 3.6 years;	• Elevated MPV levels were corelated with MACE (OR 1.67, 95% CI 1.05–2.67, p = 0.03);<br>• With a sensitivity of 48% and a specificity of 92% (AUC 0.753, p0.01), an MPV level >9.8 fL predicted MACEs;	• MPV is an independent predictor of MACE in short-term follow-up (cardiovascular death and non-fatal reinfarction within 30 days);
Monteiro Junior et al.	Prospective study	466 AMI patients (61.6 male); 70% STEMI;	• Raised MPV concentrations, in a multivariate analysis, are associated with in-hospital mortality (HR 2.97, 95% CI: 1.156.7, p = 0.024);	• MPV is an independent predictor of in-hospital mortality;

		Mean age 64.2 ± 12.8 years;		
Kurtul et al.	Retrospective single-center study	1206 STEMI patients treated by pPCI (75.3% male); Mean age 58.71 ± 13.07 years;	• In a ROC analysis, a MPV ≥ 8.65 fL (AUC 0.587) is a predictor for angiographic no-reflow phenomena and a MPV ≥ 4.7 fL is associated with higher risk of in-hospital mortality than in those with lower levels (7.3% vs 2.6%, p < 0.001);	• MPV is a predictor for short-term mortality and no-reflow phenomena;
Chunyang et al.	Retrospective observational study	1080 STEMI patients; Mean follow-up 30 months (12-68);	• High MPV/PC was an independent predictor of major adverse cardiovascular event (HR: 1.121, 95% CI: 1.056-1.190, p < 0.01), all-cause mortality (HR: 1.109, 95% CI: 1.016-1.209, p = 0.020), cardiac mortality (HR: 1.141, 95% CI: 1.038-1.253, p = 0.006), nonfatal myocardial reinfarction (HR: 1.148, 95% CI: 1.044-1.262, p = 0.004), and unplanned repeat revascularization (HR: 1.073, 95% CI: 1.007-1.144, p = 0.030);<br>• MPV/PC ratio cut-off value for predicting MACE was 0.054;	• MPV/PC ratio is a long-term adverse outcome predictor;
Ösken et al.	Retrospective cohort study	3667 STEMI patients treated by pPCI; Follow-up for five years;	• Patients with MPV/PC ratio between 0.043 and 0.357 had a 2.76 greater risk for stent thrombosis (95% CI 1.68-10.33) and 1.72 higher risk of mortality (95% CI 1.33-2.22);	• MPV/PC ratio is corelated with long-term ST and mortality;
		**TROPONINS**		
Zeljković et al.	Prospective study	220 STEMI patients (74.5% male); 12 months follow-up; Mean age 59 ± 10 years;	• Mean values of cTnT or divided into quartiles were greater in patients who developed LVEF < 50% after one year follow-up (OR 3.342 95% CI 1.17-9.52, p = 0.025; Q4: OR 6.272 95% CI 1.35-29.07, p = 0.002);	• cTnT was a predictor for LV systolic disfunction (<50%) within one year follow-up;
Mohammad et al.	Post-hoc study	578 STEMI patients; 12 months follow-up;	• A cut-off value of hs-cTnT ≤ 3500 ng/L rule out a LVEF ≤ 40% with a specificity of 59.9% and a NPV of 98.2%;	• The hs-cTnT level predicted long-term LV dysfunction;
Wanamaker et al.	Prospective study	14061 STEMI patients;	• Admission troponin was shown to be an independent predictor of in-hospital mortality, with any value beyond the reference range linked to an increased risk of death (1.8% vs 5.1% standardized difference, 18.2%);	• Regardless of baseline clinical risk, increased admission troponin levels increase mortality;
Ndrepepa et al.	Retrospective study	818 STEMI patients treated by pPCI; 3 years follow-up;	• The median preprocedural and peak postprocedural hs-cTnT levels were 153 and 1980 ng/L, respectively;	• In patients with STEMI having pPCI, admission or peak postprocedural

			• Preprocedural (adjusted HR = 1.08 95% CI (1.03–1.12), p < 0.001) and peak postprocedural hs-cTnT value (adjusted HR = 1.06 95% CI (1.04–1.08), p < 0.001) were independently linked with 3-year mortality after adjustment;	hs-cTnT is independently linked with the probability of 3-year death;
Harada et al.	Retrospective study	3783 NSTE-ACS patients; 12 months follow-up; Divided in three tertiles;	• After adjusting for other factors, postprocedural hs-TnT was found to be independently linked with the risk of all-cause death (adjusted HR=1.22 95% CI 1.13-1.33, p < 0.001 for 1-unit greater log hs-TnT);<br>• The C statistic of the model without [with baseline variables alone] and with postprocedural hs-TnT incorporation was 0.759 [0.732-0.782] and 0.772 [0.746-0.794], respectively; p < 0.001;	• Postprocedural hs-TnT is independently related with higher risk of death up to 1 year after PCI in individuals with NSTEMI who get early PCI;
**CREATINE KINASE-MB (CK-MB)**				
Johannes et al.	Observational study	1459 STEMI patients; Median follow-up 6.7 years;	• In a multivariate analysis, peak CK-MB was risk-factor for HF onset after STEMI within follow-up (HR 1.11 per 100 U/L; 95% CI 1.11 to 1.22) with a median time to HF development of 2.1 years;	• CK-MB is a risk factor for HF onset after STEMI;
Ndrepepa et al.	Retrospective study	2077 NSTE-ACS patients; Three years follow-up;	• The mortality in patients with median values of peak post-procedural CK-MB < 18.3 U L^{-1} was 18.9% compared to those with lower levels (HR 51.52, 95% CI 1.16–2.01; p < 0.001);<br>• After adjustment, peak postprocedural CK-MB (adjusted HR 51.05 CI 1.02–1.07, p < 0.001 for each 24 U L^{-1} increment) is independently associated with the risk of 3-year mortality;	• Peak post-procedural CK-MB is a predictor of three years mortality;
Hsu et al.	Prospective study	110 AMI patients; 6 months follow-up;	• Peak CK-MB (OR = 1.006 95% CI 1.001–1.011; p = 0.015) predict half-year LV remodeling;	• CK-MB is an independent predictor of LV remodeling after 6 months;
Pöyhönen et al.	Prospective study	41 AMI patients; Mean follow-up 8 years (7-9);	• Peak CK-MB had a strong correlation with chronic scar size (r = 0.83, p < 0.001) and chronic wall-motion abnormality index (WMAi) (r = 0.75, p < 0.001);	• Peak CK-MB is strongly associated with chronic scar size and WMAi;
**CYSTATIN-C (cysC)**				
Shen et al.	Retrospective study	695 STEMI patients treated by elective PCI (76.1% male); Median follow-up 3.2 years (1.9-5.1);	• Compared to low cysC group, the higher one presented a five-fold risk of composite endpoint (95% CI 3.081-7.494, p < 0.001);<br>• In a multivariate analysis, the group with higher cysC levels had 3.608 greater risk of composite endpoint (95 % CI 1.939-6.716, p < 0.001);	• Increased cysC values are associated with significant risk of composite endpoint in STEMI patients

		Mean age 58 ± 11 years;		undergoing elective PCI;
Cheng et al.	Prospective study;	218 STEMI patients treated by pPCI;	• In univariate regression (OR = 16.849, 95% CI: 4.481–63.357, p < 0.001) and in multivariate regression analysis (OR =10.07, 95% CI: 2.340–43.377, p = 0.002), higher Cys-C levels were predictors for no-reflow phenomena;<br>• The AUC of Cys-C was 0.688 (95% CI 0.557–0.780);	• CysC is a predictor for no-reflow phenomena;
Lou et al.	Retrospective cohort study;	5973 AMI patients;	• High admission cysC was corelated with elevated MACE incidence (HR = 2:293, 95% CI 1.400 to 3.755, p < 0.0001), cardiovascular mortality (HR = 3:016, 95% CI 1.694 to 5.371, p = 0.0002), and all-cause mortality (HR = 3:424, 95% CI 2.010 to 5.835, p < 0:0001);	• CysC is a predictor for MACE (cardiovascular mortality and all-cause mortality;
Brankovic et al.	Prospective study;	187 ACS patients (79% men);<br>One year follow-up;<br>Mean age 63 ± 11 years;	• Higher levels of CysC were shown to be linked with the outcome at any stage throughout the study (HR 95% CI: per 1SD increase of 2logCysC: 1.79 [1.21–2.63], p = 0.006). CysC level remained a significant predictor after adjusting for the GRACE risk score (adjusted HR 95% CI: 1.63 [1.01–2.66], p = 0.043);	• Independent of the GRACE risk score, CysC levels predict death or recurrence of ACS during the first year;
Barbarash et al.	Prospective study	357 STEMI patients;<br>Mean age 61.3 years (59.9-62.6);<br>Three years follow-up;	• Following 3 years of follow-up, serum cysC levels of 1.9 mg/l on the 12th-14th day after hospital admission were linked to a poor cardiovascular outcome in these individuals (OR 1.9 95% CI 1.2-2.9, p < 0.004);	• CysC is a predictor of adverse cardiovascular outcomes within three years follow-up;
Ma et al.	Cross-sectional study	96 ACS patients (48 STEMI, 23 NSTE-ACS, 25 UAP);<br>12-months follow-up;	• In ACS patients, blood Cys C levels (HR=1.692, 95 percent CI=1.028–2.124, P.0001) were independent influencing variables of cardiovascular events (HR=1.692, 95 percent CI=1.028– 2.124, P.0001);	• CysC is a predictor of cardiovascular events (death, ACS recurrence and HF);
Correa et al.	Double-blind, multicenter, phase 3 trial	4965 ACS patients;<br>Median follow-up 2.5 years;	• After multivariate analysis, increasing Cys-C concentration (per SD of log-transformed Cys-C) was found to be associated with a 28 % increased risk of cardiovascular death (CVD) or HF hospitalization (hazard ratio [HR] 1.28, 95 % CI 1.12-1.46, p < 0.001), including CVD (HR 1.24, % CI 1.04- 1.47, p = 0.01) and HF hospitalization (HR 1.42, 95% CI 1.19-1.69). Cys-C was linked to an increased risk of CVD, myocardial infarction, or stroke (HR 1.15, 95% CI 1.04-1.28, P0.01), including myocardial infarction (HR 1.17, 95% CI 1.02-1.33, p = 0.02);	• CysC is a predictor of adverse cardiovascular outcomes;

Mao et al.	Prospective observational study	422 NSTE-ACS patients; 12-months follow-up;	• Multivariate Cox analysis indicated that cystatin C level was an independent predictor of MACEs (HR 2.609, 95% CI 1.295–5.257, and p = 0.007);	• CysC is an independent predictor of MACE (cardiac death, non-fatal MI, TLR, HF, non-fatal stroke);
Chen et al.	Retrospective study	716 STEMI patients undergoing late PCI; Mean follow-up 40.37 months	• The high cys C group (>1.105 mg/L) had a greater long-term all-cause mortality (10.4 % vs 2.9 %, P 0.001) and a higher cardiac mortality (6.8% vs 2.1 %, p = 0.004) than the low cys C group;	• High CysC levels at admission are an independent predictor of cardiac mortality and long-term all-cause mortality;
Saito et al.	Sub-study of a multicenter, prospective, randomized controlled trial	1100 ACS patients; Median follow-up period of 4.0 years;	• In a multivariable analysis, higher levels of CysC were independent predictors of all-cause mortality (HR, 2.95, 95% CI, 1.49–5.33, p = 0.001), with the cutoff value of Cys-C to predict the occurrence of it of 1.03 mg/L;	• CysC is associated with all-cause mortality but not with other cardiovascular events in ACS patients;
**ENDOTHELIAL CELL RELATED BIOMARKERS**				
Ziaee et al.	Prospective cross-sectional study	320 ACS patients (male 73.3%); 30 days follow-up; Mean age 57.3 years;	• Higher values of endocan are corelated with 3.2-fold increased risk of MACE (95% CI 2.165-4.821, p < 0.001); • There was a positive relationship between endocan and TIMI score (r = 0.939, p < 0.001);	• Endocan was an independent predictor for MACE (in-hospital death, HF and recurrent ischemia), comparable with that of the TIMI risk score;
Dogdus et al.	Prospective cross-sectional study	137 STEMI patients (64.2% male); Mean age 56.2 ± 11.5 years;	• In a multivariate analysis, endocan was an independent predictor of the no-reflow phenomenon (p <0.001, OR 2.39, 95% CI 1.37-4.15); • A value of endocan greater than 2.7 ng/ml had 89.6% sensitivity and 74.2% specificity for the prediction of the NRP (AUC: 0.832, p < 0.001);	• Endocan was an independent predictor for no-reflow phenomenon in STEMI patients;
**ASPARTATE TRANSAMINASE (AST)**				
Gao et al.	Prospective observational study	2636 STEMI patients treated by pPCI (72% male); Two years follow-up;	• At one month, AST was associated with all-cause mortality at one month but not at two-years (HR 0.999, 95% CI 0.098–1.000, p = 0.030);	• AST levels over the 95th percentile • was linked to a higher risk of all-cause death in

		Mean age 59.5 ± 11.1 years;	• Only AST ≥ 95th percentile after adjustment for other risk factors, was corelated with all-cause mortality at two years (HR 1.796, 95% CI 0.588-5.481;	both the short and long term;
Steiniger et al.	Prospective study	1355 AMI patients; Median follow-up 8.7 years;	• After adjustments, De-Ritis ratio maintained its strong prognostic predictor role in long-term mortality (HR of 1.23 per 1-SD 95% CI: 1.07–1.42; p = 0.004);	• De-Ritis ratio was a strong independent predictor for long-term mortality;
